# Banana fruit VQ motif-containing protein5 represses cold-responsive transcription factor MaWRKY26 involved in the regulation of JA biosynthetic genes

**DOI:** 10.1038/srep23632

**Published:** 2016-03-23

**Authors:** Yu-Jie Ye, Yun-Yi Xiao, Yan-Chao Han, Wei Shan, Zhong-Qi Fan, Qun-Gang Xu, Jian-Fei Kuang, Wang-Jin Lu, Prakash Lakshmanan, Jian-Ye Chen

**Affiliations:** 1 State Key Laboratory for Conservation and Utilization of Subtropical Agro-bioresources/Guangdong Key Laboratory for Postharvest Science, College of Horticultural Science, South China Agricultural University, Guangzhou 510642, China; 2 Sugar Research Australia, 50 Meiers Road, Indooroopilly, Brisbane 4068, Queensland, Australia

## Abstract

Most harvested fruits and vegetables are stored at low temperature but many of them are highly sensitive to chilling injury. Jasmonic acid (JA), a plant hormone associated with various stress responses, is known to reduce chilling injury in fruits. However, little is known about the transcriptional regulation of JA biosynthesis in relation to cold response of fruits. Here, we show the involvement of a Group I WRKY transcription factor (TF) from banana fruit, MaWRKY26, in regulating JA biosynthesis. MaWRKY26 was found to be nuclear-localized with transcriptional activation property. *MaWRKY26* was induced by cold stress or by methyl jasmonate (MeJA), which enhances cold tolerance in banana fruit. More importantly, MaWRKY26 transactivated JA biosynthetic genes *MaLOX2, MaAOS3* and *MaOPR3* via binding to their promoters. Further, MaWRKY26 physically interacted with a VQ motif-containing protein MaVQ5, and the interaction attenuated MaWRKY26-induced transactivation of JA biosynthetic genes. These results strongly suggest that MaVQ5 might act as a repressor of MaWRKY26 in activating JA biosynthesis. Taken together, our findings provide new insights into the transcriptional regulation of JA biosynthesis in response to cold stress and a better understanding of the molecular aspects of chilling injury in banana fruit.

Low temperature is a major environmental stress that disrupts cellular homeostasis, and severely impairs plant growth and development. It is a major crop productivity constraint worldwide. Also, cold stress is experienced by most fruits and vegetables during post-harvest storage and transport. To cope with cold stress, plants have evolved sophisticated adaptive mechanisms involving altered physiological and biochemical processes, as well as reprogramming an array of stress-responsive genes, including regulatory and functional genes^1^,^2^,^3^. Among the various regulatory genes, transcription factors (TFs) play an important role in plant stress responses via acting as coordinators of stress signals and orchestrating the expression of functionalgenes^4^. Numerous cold-responsive TFs, such as C-repeat (CRT)-binding factors (CBFs)/dehydration responsive element binding factors (DREBs), ICE1 (inducer of CBFexpression 1), MYB, MYC2, EIN3, WRKY and ABRE-binding proteins/factors (AREBs/ABFs) in plants have been characterized^5^. They act through ICE-CBF transcriptional cascade or CBF-independent pathway^6^,^7^,^8^.

Among the cold-responsive TFs, the plant-specific WRKY TFs, comprise a large family of regulatory proteins. WRKY proteins contain one or two highly conserved DNA-binding domains, called WRKY domain, characterized by WRKYGQK sequences, followed by a unique zinc-finger motif of Cys and His residues at the C-terminus of these proteins^9^. Based on the number of WRKY domains and the pattern of the zinc-finger motif, WRKY proteins are divided into three major groups (I–III), with the group II further splitting into five subgroups (IIa-e)^10^,^11^. WRKY TFs are major regulatory proteins to modulate target gene expression by directly binding to W-box elements with a core sequence (C/T)TGAC(C/T) present in the promoters^11^,^12^. Since the first WRKY TF was characterized from sweet potato^13^, a large number of WRKY genes have been identified from various plants, with 72 and 109 members in *Arabidopsis thaliana* and rice, respectively^14^,^15^. WRKY TFs play important roles in plant biotic stress responses^11^,^16^,^17^ and have been implicated to abiotic stress tolerance, including cold stress^6^,^17^,^18^,^19^. For example, transgenic *Arabidopsis* plants overexpressing either soybean GmWRKY21^20^ or wheat TaWRKY10^21^ showed increased cold tolerance, while AtWRKY34 negatively mediates cold sensitivity of mature *Arabidopsis* pollen by regulating the expression of transcriptional activator CBFs^22^. Although a wide range of stress-responsive WRKY TFs have been identified and studied, the mechanistic understanding WRKY TF-mediated abiotic stress responses is progressing relatively slowly^21^,^23^. Furthermore, little information is available about WRKY TFs in non-model plants, especially in economically important fruit crops.

Phytohormones such as abscisic acid (ABA), gibberellic acid (GA), ethylene and jasmonate (JA) have been shown to modulate cold stress responses^8^. Recent studies have revealed JAs as positive regulators of cold and freezing tolerance^24^. For instance, cold stress triggers a transient increase in levels of the endogenous JA content, and exogenous application of JA significantly enhances plant cold tolerance^25^,^26^. In *Arabidopsis*, JA is perceived by the receptor CORONATINEINSENSITIVE1(COI1), an F-box protein. It degrades the JAZ (jasmonate ZIM-domain) repressors to release TFs, and initiates the expression of JA-responsive genes^27^,^28^. Among these TFs, the MYC2-clade bHLH TFs are considered as the master regulator of most JA responses, which act as activators or repressors to regulate diverse aspects of JA-mediated gene expression^29^. In addition, several WRKY TFs have been shown to be involved in JA mediated-defense responses^30^,^31^. Constitutive expression of *AtWRKY33* confers increased resistance to necrotrophic pathogens but increases susceptibility to *Pseudomonas syringae*^32^. Overexpression of *OsWRKY13* enhances rice resistance to bacterial blight and fungal blast, which is accompanied by the activation of salicylic acid (SA) biosynthesis and SA-responsive genes and the suppression of JA biosynthesis and JA-responsive genes. Interestingly, yeast-one hybrid analysis shows that OsWRKY13 binds to the promoters of its own and at least three other genes in SA- and JA-dependent biosynthesis and signaling pathways, such as *PR1a, AOS2*, and *LOX*^30^. A pair of allelic genes *OsWRKY45-1* and *OsWRKY45-2* play opposite roles in rice-bacteria interactions as OsWRKY45-1 modulates SA and JA levels whereas OsWRKY45-2 affects only JA levels^31^. These investigations demonstrate that transcriptional regulation is an important mechanism in controlling JA synthesis in response to biotic stress. However, it is unknown whether and how JA biosynthesis is transcriptionally modulated by WRKY TFs under abiotic stresses, such as cold.

Cold storage is the most common technology applied to maintain the post-harvest qualities and extend the shelf-life of many fruits. However, many tropical and sub-tropical fruits, such as bananas, are highly sensitive to chilling injury, which significantly reduces commercial quality and consumer acceptance^33^. Application of JA methyl esters, such as methyl jasmonate (MeJA) improves cold tolerance in many horticultural crops^34^,^35^,^36^,^37^. Alleviation of chilling injury in fruits by MeJA has been implicated to enhanced antioxidant enzyme activity and energy status, increased arginine, lignin accumulation, cell wall polysaccharides solubilization, a higher unsaturated/saturated fatty acid ratio, and increased production of total phenolics^34^,^35^,^36^,^37^. Our previous studies have shown that pretreatment with MeJA enhances cold tolerance in banana fruit, and several TFs, including bHLH and MYC2, are related to MeJA-induced cold tolerance via interacting with ICE-CBF regulatory pathway^33^,^38^. However, the mechanism of transcriptional regulation of JA biosynthetic genes in relation to JA-mediated cold response remains unknown. Here, we report the involvement of a Group I WRKY TF, MaWRKY26 in cold stress response of banana fruit. MaWRKY26 is cold-and MeJA-inducible, and it acts as a transcriptional activator of JA biosynthetic genes including *MaLOX2, MaAOS3* and *MaOPR3* through binding to their promoters. Further investigation show that MaWRKY26 interacts in the nucleus with a VQ motif-containing protein MaVQ5, and its transcriptional activation ability is repressed by MaVQ5. The findings presented here reveal new insights on the transcriptional regulation of JA biosynthesis in response to cold stress in banana fruit.

## Results

### Sequence analysis of MaWRKY26

**Figure 1:**
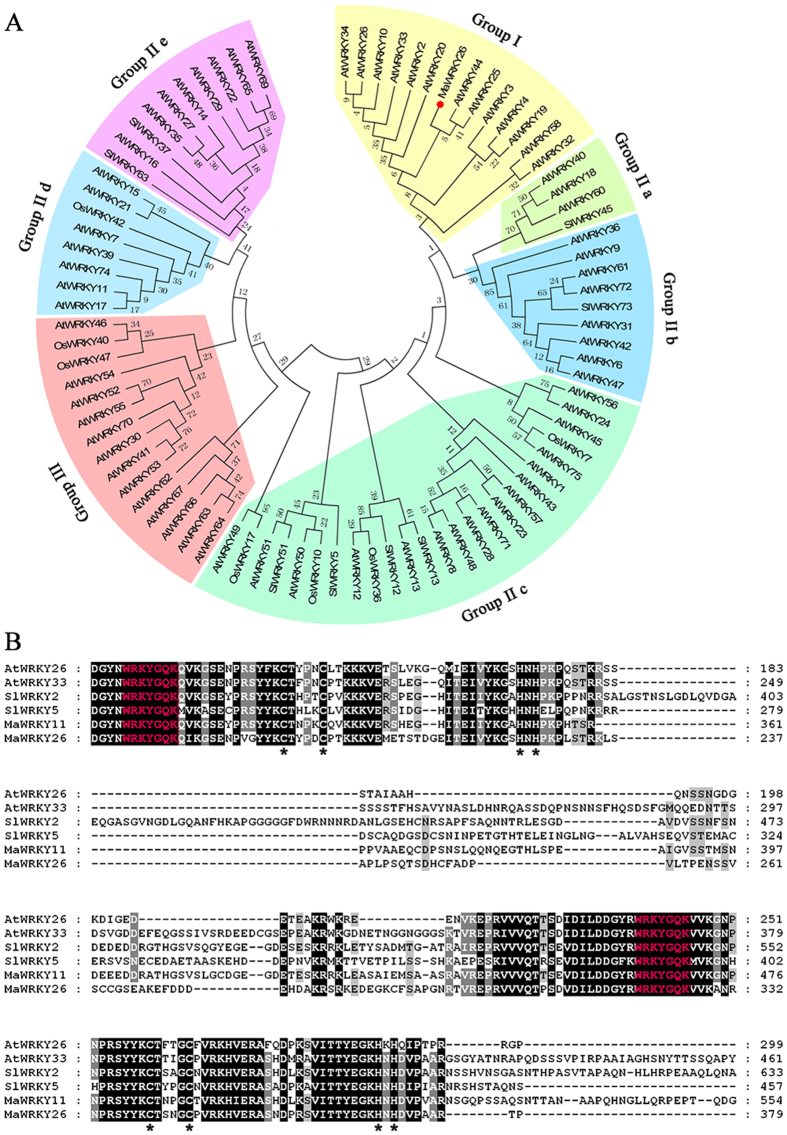
Phylogenetic analysis and multiple alignments of MaWRKY26 with other plants WRKYs. (**A**) A phylogenetic tree constructed based on WRKYs of *Arabidopsis thaliana*, tomato, rice and MaWRKY26. The WRKYs are divided into three major groups and seven sub-families, indicated by different colors. (**B**) Multiple alignments of the conserved WRKY domains between the Group I WRKYs. The WRKY motifs are shown in red letters while the zinc-finger structures are indicated by asterisks.

Based on our RNA-seq transcriptome database analysis, a putative *WRKY* gene (GSMUA_Achr3T09940_001 in the banana genome, http://banana-genome.cirad.fr/) was found induced by cold stress. Homology search showed that the gene shared the highest identity with AtWRKY26 (53%), one of the 72 WRKY TFs of *Arabidopsis thaliana*, so it was designated as *MaWRKY26* (*Musa acuminata* WRKY26). The Open Reading Frame (ORF) of MaWRKY26 is 1155 bp in length, encoding a polypeptide of 384 amino acid residues. A phylogenetic tree showed that MaWRKY26 was classified into Group I, along with AtWRKY26 and AtWRKY33 (Fig. 1A). Similar to AtWRKY26 and AtWRKY33, MaWRKY26 has two WRKY domains that contain the highly conserved amino acid sequence WRKYGQK and two putative zinc-finger motif (C-X4-CX23–24-H-X1-H) (Fig. 1B). These results clearly demonstrate that MaWRKY26 encodes a WRKY protein belonging to Group I family.

### MaWRKY26 is localized to the nucleus and possesses transcriptional activation activity

**Figure 2:**
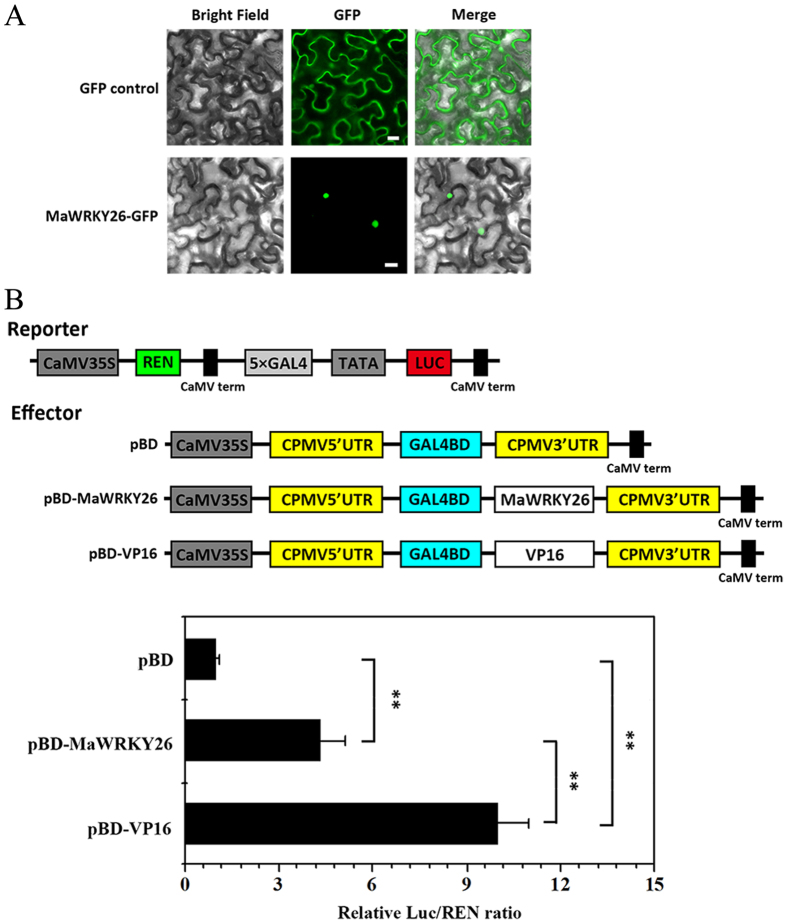
Molecular characterization of MaWRKY26. (**A**) Subcellular localization of MaWRKY26 in tobacco leaves. MaWRKY26 fused with the GFP or GFP positive control were infiltrated into tobacco leaves via *Agrobacterium tumefaciens* strain GV3101. After 48 h of the infiltration, GFP fluorescence was visualized using a fluorescence microscope. Bars, 30 μm. (**B**) Transcriptional activation ability of MaWRKY26 in tobacco leaves. The double-reporter plasmids contain 5× GAL4 and mini CaMV35S fused to firefly luciferase (LUC) and renilla luciferase (REN) driven by CaMV35S. The effector plasmids contain the MaWRKY26 or VP16 (positive control) gene fused to GAL4BD driven by the CaMV35S. The dual REN/LUC reporterand effectors were co-transformed into tobacco leaves by *Agrobacterium tumefaciens*strain GV3101. After 48 h of the infiltration, LUC and REN luciferase activities were assayed, and the transcription activation ability of MaWRKY26 is indicated by the ratio of LUC to REN. Each value represents the means of six biological replicates, and vertical bars represent the S.E. **Significant differences in values (*P* < 0.01) by Student’s t-test, compared with pBD.

A MaWRKY26-GFP fusion construct driven by the constitutive CaMV 35S promoter was used for MaWRKY26 sub-cellular localization studies. The results showed the exclusive localization of MaWRKY26-GFP in the nucleus, whereas the GFP control was observed in multiple subcellular compartments including the cytoplasm and nucleus (Fig. 2A), indicating that MaWRKY26 is a nuclear protein, possibly acting as a transcription factor.

A dual luciferase reporter plasmid harbouring five copies of the GAL4-DNA binding element and minimal TATA region of 35S promoter fused to the firefly luciferase (LUC) reporter was used to determine the *in vivo* transcriptional activity of MaWRKY26. As shown in Fig. 2B, compared with the GAL4-BD (empty, pBD) negative control, pBD-MaWRKY26 and the transcriptional activator control pBD-VP16, strongly activated the LUC reporter gene, and the LUC/REN ratio of MaWRKY26 was 3.3-fold higher than that of the negative control (Fig. 2B). These data suggest that MaWRKY26 may function as a transcriptional activator.

### *MaWRKY26* is cold- and MeJA-inducible

**Figure 3:**
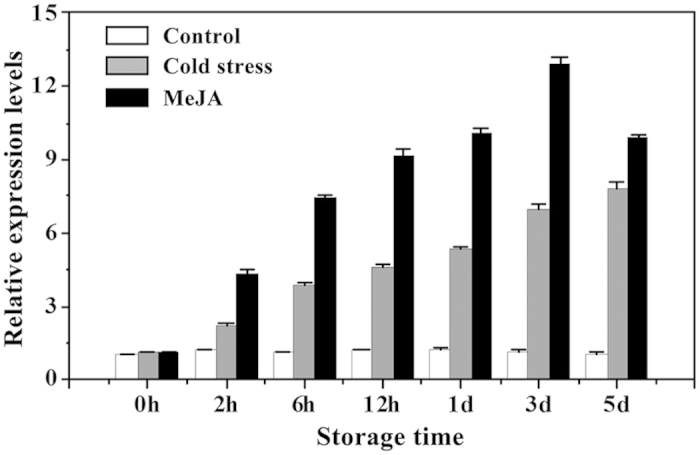
Expression of *MaWRKY26* in MeJA-treated banana fruits during 5 d of storage at 7 °C. For cold stress, fruits without MeJA pretreatment were directly stored at 7 °C, whereas for non-cold stress control, fruits were directly stored at 22 °C. Expression level at different time points was expressed as a ratio relative to the harvest time (0 d of non-cold stress control), whichwas set at 1. Each value represents the means of three biological replicates, and vertical bars indicate the S.E.

Our previous studies have shown that pre-treatment with MeJA prior to cold storage enhances cold tolerance in banana fruit^33^,^38^. To understand the possible role of MaWRKY26 in MeJA-induced banana fruit cold tolerance, expression pattern of *MaWRKY26* in the peel of cold-stored fruit after MeJA treatment was investigated by qRT-PCR. As shown in Fig. 3, *MaWRKY26* was cold-inducible, and its transcript levels increased rapidly following MeJA treatment during cold storage. Compared with the expression of MaWRKY26 in control fruits (no cold treatment), the expression of MaWRKY26 in the fruit directly stored at 7 °C (cold stress) increased after 2 h and reached ~8-fold on day 5 (Fig. 3). In MeJA-treated fruit MaWRKY26 transcript level increased rapidly within 2 h, reaching 1.88- and 1.86-fold higher than that in the cold stressed fruit after 1 and 3 d of storage, respectively (Fig. 3). These data indicate that *MaWRKY26* is cold- and MeJA-inducible, suggesting its possible involvement in MeJA-induced cold tolerance of banana fruit.

### MaWRKY26 specifically binds to the promoters of JA biosynthetic genes

**Figure 4:**
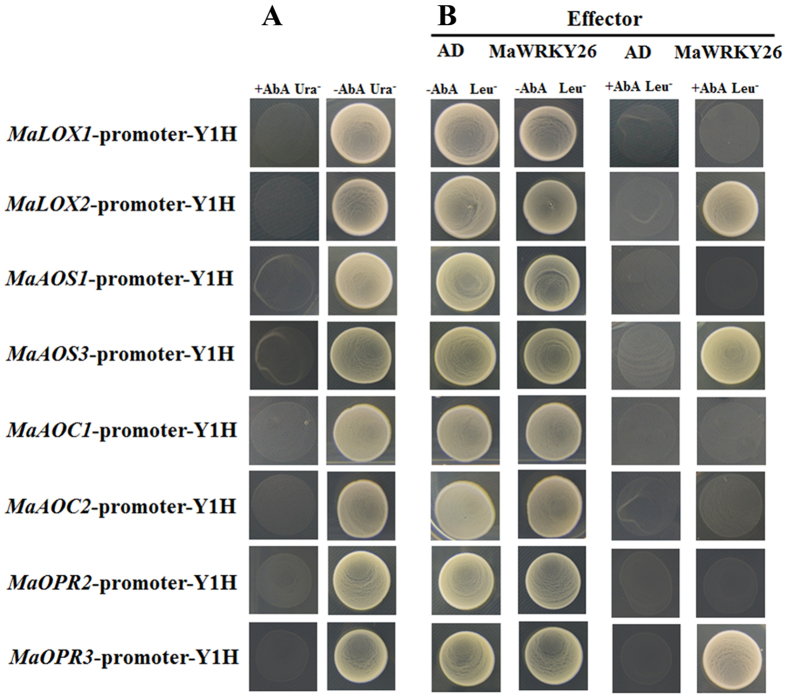
Yeast one-hybrid (Y1H) showing the association of MaWRKY26 with promoters of JA biosynthetic genes. The promoters of eight JA biosynthetic genes including *MaLOX1, MaLOX2, MaAOS1, MaAOS3, MaAOC1, MaAOC2, MaOPR2* and *MaOPR3*, were cloned in front of the reporter gene AUR1-C, an antibiotic resistance gene that confers Aureobasidin A (AbA) resistance in yeast. (**A**) No basal activities of *MaLOX1, MaLOX2, MaAOS1, MaAOS3, MaAOC1, MaAOC2, MaOPR2* and *MaOPR3* promoters was detected in yeast grown on SD medium lacking Leu in the presence of AbA. (**B**) Yeast growth assays after the yeast Y1H reporter strains harboring the promoters were transformed with plasmids carrying cassettes constitutively expressing MaWRKY26 effector or empty (AD, negative control). Interaction was determined based on the ability of transformed yeast to grow on SD medium lacking Leu in the presence of AbA.

It is well established that WRKY TFs preferentially bind to the so-called W-box elements with a core sequence (C/T)TGAC(C/T) in their target promoters^11^. Sequence analysis identified W-box (TTGAC) elements in the promoters of JA biosynthetic genes, including *MaLOX1, MaLOX2, MaAOS1, MaAOS3, MaAOC1, MaACO2, MaOPR2* and *MaOPR3* (Supplementary Text S1), suggesting that these JA biosynthetic genes might be the direct targets of MaWRKY26. Yeast one-hybrid (Y1H) assay was first carried out to examine the binding of MaWRKY26 to the promoters of JA biosynthetic genes. As shown in Fig. 4A, no basal activities of JA biosynthetic gene promoters were detected in yeast. After the Y1H reporter strains were transformed with plasmids carrying cassettes constitutively expressing MaWRKY26 effector, yeast cells harboring *MaLOX2, MaAOS3* or *MaOPR3* promoter grew well in the presence of AbA, while yeast cells harboring other promoters did not (Fig. 4B), indicating that MaWRKY26 can bind to *MaLOX2, MaAOS3* or*MaOPR3* promoter in yeast.

**Figure 5:**
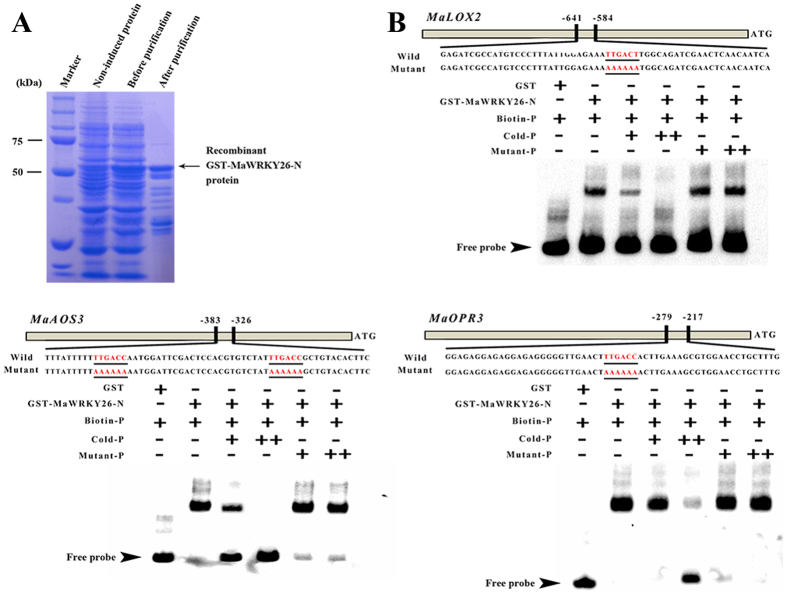
Electrophoretic mobility shift assay (EMSA) showing the association of MaWRKY26 with promoters of JA biosynthetic genes such as *MaLOX2, MaAOS3*, and MaOPR3. (**A**) SDS–PAGE gel stained with Coomassie blue demonstrating affinity purification of the recombinant MaWRKY26 protein used for the EMSA. (**B**) MaWRKY26 binds directly to the promoters of *MaLOX2, MaAOS3*, and *MaOPR3* containing W-box (TTGAC) element. Biotin-labeled DNA probe from the promoters or mutant probe was incubated with GST-MaWRKY26-N protein, and the DNA-protein complexes were separated on 6% native polyacrylamide gels. + or ++ indicate increasing amounts unlabeled probes for competition.

A DNA electrophoretic mobility-shift assay (EMSA) was conducted to further validate the interaction of MaWRKY26 with the *MaLOX2, MaAOS3* or *MaOPR3* promoter. DNA fragment containing the W-box elements in the promoter region of *MaLOX2, MaAOS3* or *MaOPR3* was used as probe. Recombinant glutathione S-transferase (GST)-MaWRKY26 fusion protein was expressed and purified from *Escherichia coli* (Fig. 5A). The recombinant MaWRKY26 was able to bind the *MaLOX2, MaAOS3* or *MaOPR3* promoter fragment and caused mobility shifts, and the binding was abolished by increasing the amount of unlabeled competitors with the same sequence, but not by the mutated probes (Fig. 5B). In addition, the mobility shift was not seen when the *MaLOX2, MaAOS3* or *MaOPR3* promoter fragment was incubated with GST alone (Fig. 5B), implying that MaWRKY26 specifically binds to the promoters of *MaLOX2, MaAOS3* and *MaOPR3*. It is important to note that similar to MaWRKY26, the expression of *MaLOX2, MaAOS3* and *MaOPR3* were also cold- and MeJA-inducible (Supplementary Figure S1). Collectively, these data indicate that MaWRKY26 might be regulating JA biosynthesis via transcriptional regulation of JA biosynthetic genes, such as *MaLOX2, MaAOS3* and *MaOPR3*.

### MaWRKY26 physically interacts with MaVQ5

**Figure 6:**
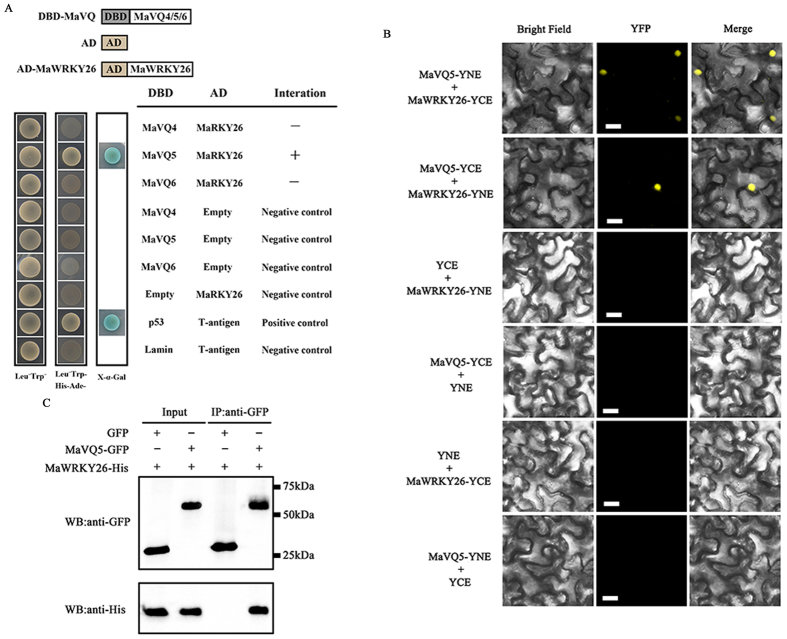
MaWRKY26 interacts with MaVQ5 *in vitro* and *in vivo*. (**A**) Yeast two-hybrid (Y2H) assay for the interaction between MaWRKY26 and MaVQ5. The coding regions of MaVQs and MaWRKY26 were fused with DBD and AD vectors, respectively, as indicated, and co-transformed into the yeast strain Gold Y2H. The ability of yeast cells to grow on synthetic medium lacking tryptophan, leucine, histidine and adenine but containing 125 μm Aureobasidin A, and to turn blue in the presence of the chromogenic substrate X-α-Gal, was scored as a positive interaction. (**B**) Bimolecular fluorescence complementation (BiFC) in tobacco leaf epidermal cells showing the interaction between MaWRKY26 and MaVQ5 in living cells. MaWRKY26 fused with the N-terminus of YFP and MaVQ5 fused with the C-terminus of YFP, or MaVQ5 fused with the N-terminus of YFP and MaWRKY26 fused with the C-terminus of YFP, were co-transfected into tobacco leaves and visualized using confocal microscopy. Expressions of MaWRKY26 or MaVQ5 alone were used as negative controls. YFP-fluorescence of YFP; Merge-digital merge of bright field and fluorescent images. The length of the bar indicated in the photographs is 30 μm. (**C**) Co-IP assay showing the interaction between MaWRKY26 and MaVQ5. Tobacco leaves co-expressing MaVQ5-GFP and MaWRKY26-His, or empty-GFP and MaWRKY26-His, was used to immunoprecipitate with the anti-GFP antibody, and the immunoblot was probed with the anti-GFP and anti-His antibody, respectively.

Previous studies have shown that VQ proteins interact with several members of Group I and Group IIc WRKY TFs to mediate development or stress responses, such as pathogens and salt^39^,^40^,^41^. Six VQ domain-containing genes, numbered *MaVQ1* to *MaVQ6*, were found in the banana genome (Supplementary Figure S2). To investigate whether MaVQs interact with MaWRKY26, the GAL4 transcription activation-based yeast two-hybrid system was used. *MaVQ4/5/6* were fused with the GAL4 DNA-binding domain (DBD-MaVQs) and *MaWRKY26* was ligated with the activation domain (AD-MaWRKY26) to create the bait and prey since MaWRKY26 and MaVQ1/2/3 showed transcription-activating activities in yeast (data not shown). As shown in Fig. 6A, co-expression of DBD-MaVQ5 with AD-MaWRKY26 caused strong activation of the α-galactosidase activity, indicating that MaVQ5 interacts with MaWRKY26 in yeast cells; the other MaVQs did not do so.

The interaction between MaWRKY26 and MaVQ5 in plant cells was further substantiated by bimolecular fluorescence complementation (BiFC) assay. Both MaWRKY26 and MaVQ5 were fused to the N-terminal 174-amino acid portion of yellow fluorescent protein (YFP) as well as the C-terminal 66-amino acid portion of YFP in the pEAQ vector. As shown in Fig. 6B, co-expression of MaWRKY26-YNE and MaVQ5-YCE, or MaWRKY26-YCE and MaVQ5-YNE reconstituted a functional YFP in the nucleus, whereas co-expression with the negative control combinations failed to generate YFP fluorescence. As well as BiFC assay, the MaWRKY26-MaVQ5 interaction was verified by co-immunoprecipitation (Co-IP) assay using plant total protein. The *MaVQ5* and *MaWRKY26* were inserted into pEAQ-HT-GFP and pEAQ-HT-His vector, respectively (Fig. 6C), which have been shown to be a versatile expression system for easy and quick transient expression of heterologous proteins in plants^42^. As expected, the MaWRKY26-His fusion protein can be detected after immunoprecipitation of MaVQ5-GFP using anti-GFP antibody microbeads (Fig. 6C). Collectively, these data demonstrate that MaWRKY26 physically interacts with MaVQ5. In addition, MaVQ5 was also localized to the nucleus, and was cold-responsive (Supplementary Figure S3).

### MaVQ5 decreases MaWRKY26 trans-activation of JA biosynthetic genes

**Figure 7:**
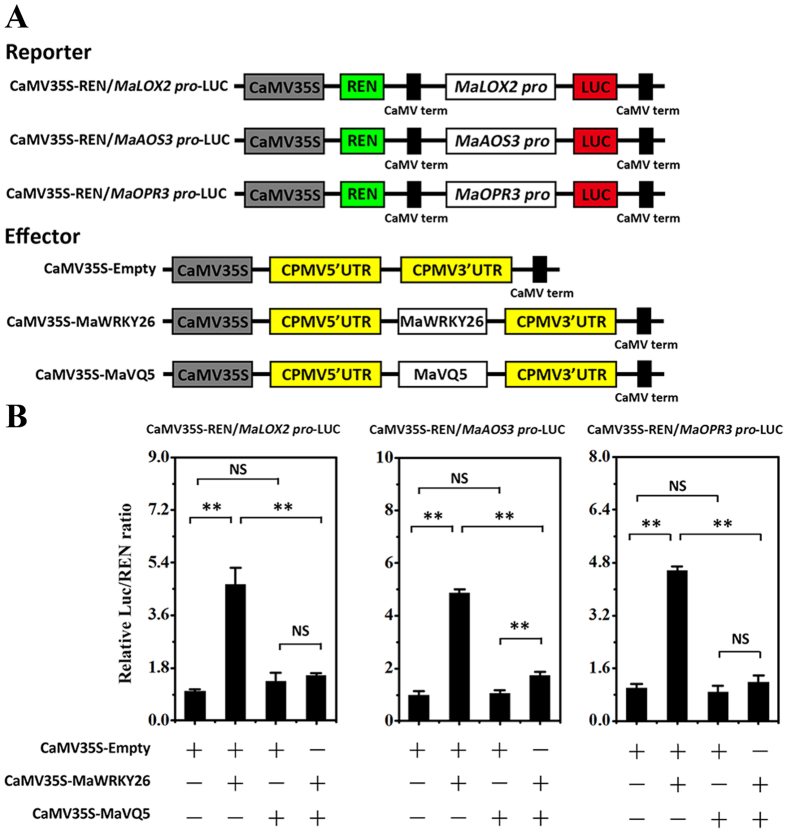
Transient dual-luciferase reporter assays showing MaVQ5’s ability to repress MaWRKY26’s transcription activation of *MaLOX2, MaAOS3* or *MaOPR3* promoter. (**A**) Constructs used in the transient transactivation assays. (**B**) *Agrobacterium* strain GV3101 carrying the LUC reporter plasmid and different combinations of effector plasmids was infiltrated into *N. benthamiana* leaves, and the luciferase activity at the site of infiltration was measured 3 d after infiltration. The activities of firefly luciferase (LUC) and renilla luciferase (REN) were measured sequentially, and the LUC/REN ratio was calculated as the final transcriptional activation activity. Data represent the mean ± S.E. of six biological replicates. Asterisks indicate significant difference (*P* < 0.01) between combinations by Student’s t-test.

The physical interaction between MaWRKY26 and MaVQ5 led us to investigate whether or not MaVQ5 interferes with transcriptional activation of JA biosynthetic genes by MaWRKY26. To this end, we performed transient expression assays using the dual-luciferase reporter system. In this experiment, the *MaLOX2, MaAOS3* or *MaOPR3* promoter-driven LUC (*MaLOX2, MaAOS3* or *MaOPR3* pro-LUC) and CaMV35S promoter-driven REN (CaMV35S-REN; as an internal control) were constructed in the same plasmid, together with an effector plasmid expressing MaWRKY26 or MaVQ5 (Fig. 7A), and expressed in the leaves of *N. benthamiana*. The LUC/REN ratio, which reflects *in vivo* MaWRKY26 transcriptional activity, when MaWRKY26 and/or MaVQ5 were co-expressed, was monitored. Compared with the control that was co-transfected with the empty construct, co-expression of MaWRKY26 with *MaLOX2, MaAOS3* or *MaOPR3* pro-LUC significantly increased the LUC/REN ratio (Fig. 7B), suggesting that MaWRKY26 trans-activated JA biosynthetic genes. However, the trans-activation was decreased when MaVQ5 was co-expressed (Fig. 7B). These results demonstrate that MaVQ5 likely acts as a repressor of MaWRKY26 in its trans-activation of JA biosynthetic genes.

## Discussion

WRKYs, one of the largest families of transcriptional regulators, are found exclusively in plants. They are distributed across different species, with 72 representatives in *Arabidopsis*, and more than 100 members in rice, soybean or poplar^11^,^18^. WRKY proteins are important components of a plant signaling web that regulates various physiological and developmental processes^18^. However, only a small number of WRKYs have been functionally characterized, which are predominantly in the model plants, while the biological roles and the underlying mechanisms of many WRKYs, particularly in economically important fruits, remain unknown. Elucidating the functional aspects of WRKYs in fruits will improve our understanding of the diverse roles of this TFs. In this study, we show that MaWRKY26 of banana fruit maybe play a positive role in response to cold stress by regulating JA biosynthetic genes *LOX, AOS* and *OPR*.

The defining character of WRKYs is the presence of a DNA binding domain containing the conserved WRKYGQK sequences and anatypical zinc-finger motif, based on which the WRKYs are divided into three major groups^10^,^11^. MaWRKY26 contained two WRKY domains consisting of a highly conserved WRKYGQK motifand two putative zinc-finger motifs (C-X4-CX23–24-H-X1-H) (Fig. 1A). According to these unique signatures, MaWRKY26 is classified into Group I category. This classification is supported by the phylogenetic tree, in which MaWRKY26 was clustered in the same clad with *Arabidopsis* AtWRKY20, AtWRKY25, AtWRKY26, AtWRKY33, AtWRKY34 and etc (Fig. 1B). Among the Group I WRKYs, AtWRKY33 has been extensively studied and shown to play critical roles in plant immune systems against necrotrophic pathogens and plant’s responses to abiotic stresses, such as salt and heat^43^. In addition, AtWRKY25, AtWRKY26, and AtWRKY33 have been implicated in plant thermotolerance^44^. In *Arabidopsis*, 8 cold-responsive WRKYs are reported^45^ and the AtWRKY34 reduces cold sensitivity of mature *Arabidopsis* pollen^22^, although the exact mechanism underpinning this response is unclear. In our study *MaWRKY26* was found cold-inducible and its transcript levels increased rapidly following MeJA treatment during cold storage (Fig. 3), indicating that MaWRKY26 might act as a positive regulator of cold response. Our results together with previous findings suggest a significant role for Group I WRKY members in biotic and abiotic stress responses. They might function as a node of convergence that integrates biotic and abiotic stress signals and thus hold great potential for improving stress tolerance, though the exact mechanism of action remains unclear. It is, therefore, important to identify their downstream targets.

So far, a number of downstream target genes of different WRKY members have been identified, and WRKYs act as transcriptional activators or repressors of those genes. For examples, *Arabidopsis* WRKY28 and WRKY46, that are rapidly induced by pathogen elicitors, regulate SA-mediated systemic acquired resistance via the activation of SA biosynthetic genes *ICS1* and *PBS3*, respectively^46^. A number of WRKY TFs are implicated in the regulation of ABA biosynthesis and signaling. For instance, AtWRKY57 directly binds the W-box of a stress-responsive gene *RD29A* and the promoter of ABA biosynthesis gene *NCED3*, suggesting its involvement in modulating ABA level and water stress response in *Arabidopsis*^47^. AtWRKY41 acts as a negative regulator of *Arabidopsis* seed germination via direct regulation of genes involved in ABA signaling^17^,^48^, while AtWRKY33 negatively regulates ABA levels by directly targeting and repressing *NCED3* and *NCED5* expression, which is critical for *Arabidopsis* immunity towards *Botrytis cinerea* 2100^49^. Collectively, these results reveal that WRKYs function in stress responses through positive or negative regulation of different hormone biosynthesis and signaling pathways. The phytohormone JA is also an important regulatory signal influencing plant stress responses. In *Arabidopsis*, under cold stress, expression of JA biosynthetic gene was upregulated, leading to JA accumulation, and exogenous application of MeJA enhances cold tolerance^26^. Similarly, our previous study shows that MeJA treatment induces expression of JA biosynthetic genes and increases endogenous JA level during cold storage, and reduces cold injury in banana fruit^33^. Previous studies have described the important role of WRKY TFs in JA signaling and JA mediated-defense response. For instance, overexpression of *VvWRKY1* in grapevines induces expression of JA pathway-related genes and confers improved tolerance to downy mildew^50^. The *Arabidopsis* WRKY33 negatively regulates the *JAZ1* and *JAZ5* transcripts by binding their promoter regions^32^, while soybean GbWRKY1 directly activates *JAZ1* expression^51^. However, whether, and if so how, JA biosynthesis is transcriptionally modulated by WRKY TFs under abiotic stress such as cold stress is largely unknown. In the present work, although the eight JA biosynthetic genes contain the conserved W-box elements, using Y1H, EMSA and transient expression assays, we showed that MaWRKY26 specifically bound to W-box present in the promoters of three JA biosynthetic genes, *MaLOX2, MaAOS3* and *MaOPR3*, and transactivated their expressions (Figs 4, 5 and 7). Similarly, previous studies indicate that although the W-box elements is required for specific DNA binding, additional adjacent DNA sequences outside the W-box elements also partly determine binding site preference^11^,^17^,^18^,^19^. A recent study also demonstrated that TCP TFs positively regulate JA synthesis by directly binding to the JA biosynthetic gene *LOX2* during the immune response in *Arabidopsis thaliana*^52^. These results suggest that transcriptional regulation is a pivotal mechanism in controlling JA biosynthesis under stress conditions. Intriguingly, WRKY proteins can activate or repress the expression of genes, depending on the nature of the target promoter sequence^11^,^53^,^54^. Very recently, through ChIP-seq and RNA-seq analyses, 1576 target genes of *Arabidopsis* WRKY33 were identified, and it possesses dual functionality acting either as a repressor or as an activator in a promoter-context dependent manner following pathogen challenge^49^. Therefore, genome-wide identification of MaWRKY26 targets would be helpful to confirm whether MaWRKY26 also acts as a dual-function TF, as well as reveal the regulatory network underpinning cold stress response governed by MaWRKY26.

Several different types of proteins like MAP kinases, MAP kinase kinases and VQ proteins, have been shown to interact with WRKY TFs, resulting in activation or repression of DNA-binding and transcriptional regulation activities of WRKYs^18^,^19^,^55^,^56^. VQ proteins are a group of cofactors containing a short VQ-related motif (FxxxVQxLTG). In *Arabidopsis*, AtWRKY25 and AtWRKY33 form complexes with a VQ protein, MKS1 (MAP KINASE SUBSTRATE1) that also interacts with and acts as a substrate of MAPK4^57^,^58^. AtWRKY33 also interacts with two other VQ proteins in the nucleus, SIGMA FACTOR-INTERACTING PROTEIN1 (SIB1) and SIB2^39^. MKS1, SIB1, and SIB2 bind to the C-terminal WRKY domain and stimulate the DNA-binding activity of AtWRKY33, indicating that they function as activators of AtWRKY33 in plant defense against necrotrophic pathogens^39^. However, VQ proteins also appear to act as repressors of defense-related genes via their interaction with WRKYs. Indeed, the protein VQ4/MVQ1 functions as a negative regulator of WRKY-type transcriptional activators including WRKY33, showing the complexity of WRKY TFs regulation^59^. In the current study, we identified a new partnership between MaWRKY26 and MaVQ5 associated with MeJA-mediated cold stress response of banana fruit. Our results showed that MaWRKY26 interacted with MaVQ5 (Fig. 6), and this interaction attenuated the transcriptional activation of JA biosynthetic genes by MaWRKY26 (Fig. 7B), demonstrating that MaVQ5 may be acting as a repressor and antagonistically with MaWRKY26 in relation to MeJA-mediated cold stress response.

Post-translational modifications, such as phosphorylation and ubiquitination play important roles in WRKY processes. In tobacco, a Group I WRKY protein NtWRKY1 is phosphorylated by SALICYLIC ACID INDUCED PROTEIN KINASES (SIPK), which leads to increased DNA-binding activity to a W-box-containing DNA molecule^60^. AtWRKY53 is polyubiquitinated by a HECT domain E3 ubiquitin ligase UPL5, which leads to its degradation during leaf senescence^61^. Recent research shows that jasmonate triggers JAV1 degradation via the 26S proteasome, liberating its potential interactors WRKY28 and WRKY51 to activate defensive gene expression and resistance against insects and pathogens^62^. In *Arabidopsis* protoplast assays, stimulation by pathogen-associated molecular patterns (PAMPs) resulted in degradation of VQ4/MVQ1 following MAPK-mediated phosphorylation, enabling WRKY33 to activate transcription of a defense-related reporter gene^59^. Similarly, whether MeJA treatment induces the degradation of MaVQ5 to liberate MaWRKY26 to activate JA biosynthetic genes, needs to be established.

In summary, a novel Group I WRKY TF, MaWRKY26, is identified from banana fruit. MaWRKY26 is a nuclear protein, and may function as a transcriptional activator. *MaWRKY26* is cold-inducible and its expression is enhanced by MeJA treatment, which induces cold tolerance in banana fruit. More importantly, MaWRKY26 transactivates JA biosynthetic genes *MaLOX2, MaAOS3* and *MaOPR3* via binding to their promoters, and this activation is antagonized by MaVQ5, which physically interacts with MaWRKY26. Collectively, our findings provide new insights into the transcriptional regulation of JA biosynthesis in response to cold stress and cold tolerance.

## Materials and Methods

### Plant materials and treatments

Pre-climacteric banana (*Musa acuminata* AAA Group, cv. Cavendish) fruit at the 75–80% maturation (about 12 weeks after anthesis) were harvested from a local commercial plantation near Guangzhou, China, and transported immediately to the laboratory. Each banana hand was cut into individual fingers. Banana fruit of uniform weight, shape and maturity, and free of visual defects, were used for this study. Fruits were treated with MeJA (100 μM) as described previously^33^, and the treated fruits were stored immediately at 7 °C (cold stress) and 90% RH for 5 d. For cold stress, fruits without MeJA pretreatment were directly stored at 7 °C, while for non-chilling temperature control, fruits were directly stored at 22 °C. Samples were taken at 0, 6 and 12 h and 1, 3, 5 d from the start of the experiment, and banana peel was collected. The sampled peel tissues were cut into small pieces, frozen in liquid nitrogen and stored at −80 °C for further use.

### RNA extraction, gene isolation, and sequence analysis

Frozen banana peel was ground in liquid nitrogen using a mortar and pestle. Total RNA was extracted using the hot borate method of Wan and Wilkins^63^, and the extract was treated with DNAse I digestion using an RNAse-free kit (Promega Madison, WI, USA). The DNA-free total RNA was used as template for reverse-transcription PCR. The first-strand cDNA of the product was applied to PCR amplification.

Based on our transcriptome database obtained by RNA-seq analysis, a genome segment with high sequence homology toWRKY26, termed *MaWRKY26* (GSMUA_Achr3T09940_001 in the banana genome, http://banana-genome.cirad.fr/) was found to be up-regulated by cold stress. This segment was cloned and sequenced. In addition, the sequences of JA biosynthetic genes, including *MaLOX1, MaLOX2*,*MaAOS1, MaAOS3, MaAOC1, MaAOC2, MaOPR2* and *MaOPR3*, were also obtained from Banana Genome Hub (http://banana-genome.cirad.fr/)^64^. These sequences were verified by further cloning from banana fruit peel and sequencing (primers are listed in Supplementary Table S1). Gene sequence was subjected to homology search in the NCBI (National Center for Biotechnology Information) database. Multiple alignments were analyzed by CLUSTALW (version 1.83) and GeneDoc software, and a phylogenetic tree of WRKY proteins was constructed using the UPGMA method in the MEGA5.

### Quantitative real-time PCR analysis

For quantitative real-time PCR (qRT-PCR), the gene-specific oligonucleotide primers used are described in Supplementary Table S1. The length of qRT-PCR products ranged from 80 to 300 bp. Quality and specificity of each pair ofprimers were proved by melting curves and product resequencing. The *MaACT1* was used as the reference gene for monitoring the abundance of the mRNA accordingto our previous study on the selection of reliable reference genes under different experimental conditions^65^. qRT-PCR was carried out on a Bio-Rad CFX96 Real-Time PCR System using the SYBR^®^Green PCR Supermix Kit (Bio-Rad Laboratories) following the manufacturer’s instructions. All qRT-PCR reactions were normalized using Ct value corresponding to the reference gene. The relative expression levels of target gene were calculated with the formula 2^−ΔΔCT^.

### Subcellular localization assay

Vectors for subcellular localization assay were constructed by sub-cloning the coding regions of *MaWRKY26* and *MaVQ5* into the pEAQ-HT-GFP vector (kindly supplied by Dr. George P. Lomonossoff), resulting in MaWRKY26-GFP and MaVQ5-GFP, respectively. Primers used are shown in Supplementary Table S1. The fusion constructs and control vector were electroporated into *Agrobacterium tumefaciens* strain GV3101 using Gene PulserXcell^TM^ Electroporation Systems (Bio-Rad, CA). Tobacco (*Nicotiana benthamiana*) leaf infiltration assay for sub-cellular localization was performed as described previously^66^. After infiltration, plants were incubated at 22 °C with 16 h photoperiod for at least 2 days before analysis. GFP fluorescence signals were observed with a fluorescence microscope (Zeiss Axioskop 2 Plus). All transient expression assays were repeated at least three times.

### Isolation of JA biosynthetic gene promoters and yeast one-hybrid (Y1H) assay

Genomic DNA was extracted from banana leaves using the DNeasy PlantMini Kit (Qiagen). The promoters of JA biosynthetic genes including *MaLOX1, MaLOX2*,*MaAOS1, MaAOS3, MaAOC1, MaAOC2, MaOPR2* and *MaOPR3*, were isolated using a Genome Walker Kit (Clontech) with nested PCR according to the manufacturer’s instructions. Gene-specific primers for the nested PCR arelisted in Supplementary Table S1. Conserved *cis*-element motifs of promoter were predicted using PLACE (http://www.dna.affrc.go.jp/PLACE/signalscan.html) and Plant-CARE (http://bioinformatics.psb.ugent.be/webtools/plantcare/html/) databases.

Y1H assay was performed as described in our previous publications^67^,^68^, using the Matchmaker^TM^ Gold Yeast One-Hybrid System (Clontech, CA). The promoters of JA biosynthetic genes (Supplementary Text S1) were sub-cloned into pAbAi. The resultant plasmids were linearized and transformed into Y1H Gold strainby following the manufacturer’s instructions. Positive yeast cells were then transformed with pGADT7-AD, which contained the effector MaWRKY26. The DNA-protein interaction was determined based on the growth ability of the co-transformants on SD/-Leu medium with Aureobasidin A (AbA) according to the manufacturer’s protocol. The primers used in Y1H assay are listed in Supplementary Table S1.

### Protein expression and electrophoretic mobility shift assay (EMSA)

The coding region of *MaWRKY26* was PCR-amplified and cloned into pGEX-4T-1 (Amersham Biosciences) to fuse in frame with GST. The resulting construct was transformed into *Escherichia coli* strain BM Rosetta (DE3). Protein expression was induced in a 500-mL culture using 0.5 mM isopropyl β-D-1-thiogalactopyranoside (IPTG), and cells were collected 16 h after induction at 28 °C. The recombinant protein was purified using a Pierce GST spin purification kit (Thermo Scientific) following the instructions. The resulting protein was checked for size and purity by SDS-PAGE and Coomassie Brilliant Blue staining. Protein concentration was determined using a RC/DC protein assay kit, based on the Lowry assay (Bio-Rad).

The fragments of ~60 bp containing putative WRKY binding region in the promoters of JA biosynthetic genes were amplified and labeled with biotin at the 5′ end. EMSA was performed using a LightShift Chemiluminescent EMSA kit (Thermo Scientific), as previously described in our recent report^68^. Briefly, the binding reaction mixture (20 μL) contained 25 mM HEPES-KOH (pH 7.5), 100 mM KCI, 0.1 mM ethylene diaminetetra acetic acid (EDTA), 17% glycerol, 1 mM DTT, 50 ng of purified protein, ~1 pmol of labelled probe, competitor DNA (25, 100 or 400 pmol), and 4 mg of poly (dI-dC) to minimize nonspecific interactions. The assay mixtures were incubated for 25 min at 22 °C. Biotin-labeled DNA was detected by the chemiluminescence method according to the manufacture’s protocol on a ChemiDoc™ MP Imaging System (Bio-Rad). The primers used in the EMSA assay are listed in Supplementary Table S1.

### Yeast two-hybrid (Y2H) analysis

Y2H assay was performed using the Matchmaker™ Gold Yeast Two-Hybrid Systems (Clontech), as previously described^68^. The coding sequences of *MaVQ4/5/6* and *MaWRKY26* were subcloned into pGBKT7 or pGADT7 vector to fuse with the DNA-binding domain (DBD) and activation domain (AD), respectively, to create the bait and prey (primers are listed in Supplementary Table S1). The bait and prey constructs were co-transformed into yeast strain Gold Y2H by the lithium acetate method, and yeast cells were grown on DDO medium (minimal media double dropouts, SD medium with -Leu/-Trp) for 3 d according to the manufacturer’s protocol. Transformed colonies were plated onto QDO medium (minimal media quadruple dropouts, SD medium with -Leu/-Trp/-Ade/-His), QDO media containing 4 mg mL-1 X-α-Gal (α-Gal) for blue color development, to test the possible interaction between MaVQ5 and MaWRKY26 according to their growth status and the activity of α-galactosidase.

### Bimolecular fluorescence complementation (BiFC) assay

To create constructs for BiFC assay, the full-length coding sequences of *MaWRKY26* and *MaVQ5* without their stop codons in fusion with YNE and YCE, respectively, were cloned into the pEAQ-HT vector^66^. The resulting constructs were then introduced into the *Agrobacterium tumefaciens* strain GV3101, and co-infiltrated into tobacco (*Nicotiana benthamiana*) leaves as described above. Infected tissues were analyzed at 48 h after infiltration. YFP fluorescence was captured using the Confocal Spectral Microscope Imaging System (Leica TCS SP5), with an argon blue laser at 488 nm, a beam splitter for excitation at 500 nm, and a spectral detector set between 515 nm and 540 nm. Primers used for generating the constructs are listed in Supplementary Table S1.

### *In vivo* coimmunoprecipitation (Co-IP) assay

To create MaWRKY26-His and MaVQ5-GFP constructs, full-length *MaWRKY26* or *MaVQ5* was obtained by PCR amplification and cloned into pEAQ-HT-His and pEAQ-HT-GFP vectors, respectively^66^. Primers used are shown in Supplementary Table S1. And then the resulting constructs were introduced into *Agrobacterium tumefaciens* strain GV3101 and infiltrated into the abaxial side of 4- to 6-wk-old tobacco (*Nicotiana benthamiana*) leaves using a 1-mL needleless syringe. *In vivo* Co-IP assay was performed as described byYang *et al*.^69^. For proteasome inhibition, leaves were infiltrated with 10 mM MgCl_2_ and 50 mM MG132 (Sigma-Aldrich) solution for 4 h before sample collection. 48 hours after infiltration, tobacco leaves were ground in liquid nitrogen. Proteins were extracted using an extraction buffer (50 mM Tris-HCl, pH = 7.4, 150 mM NaCl, 2 mM MgCl_2_, 20% glycerol, 5 mM DTT, and 0.1% Nonidet P-40) including a protease inhibitor cocktail. Tobacco cell debris was pelleted by centrifugation at 13,000 rpm for 12 min under 4 °C. The supernatant was incubated with 10 μl of anti-GFP antibody (Abcam) at 4 °C overnightto capture the epitope-tagged protein. The second day, 50 μl of protein A agarose beads (Roche) was added. After 4 h of incubation at 4 °C, the beads were centrifuged and washed three times using a washing buffer (50 mM Tris-HCl, pH = 7.4, 150 mM NaCl, 2 mM MgCl_2_, 10% glycerol, 5 mM DTT, and 0.1% Nonidet P-40). The immunoprecipitated protein complex was eluted with 40 μl of 2.5× SDS sample buffer, separated by SDS-PAGE and then subjected to western blotting analysis using 4000-fold diluted anti-His antibody (Abcam) and anti-GFP antibody (Abcam), respectively, with secondary goat anti-rabbit IgG peroxidase antibody (Thermo Scientific). Detection was carried out using the chemiluminescent substrate SuperSignal West Pico (Thermo Scientific) for horse-radish peroxidase and imaged on a ChemiDoc™ MP Imaging System (Bio-Rad Laboratories).

### Dual-luciferase transient expression assay

The double reporter vector includes a native GAL4-LUC (firefly luciferase), and an internal control REN (renilla luciferase) driven by the CaMV35S promoter, which was modified based on the pGreenII 0800-LUC reporter vector^70^. GAL4-LUC contains five copies of the GAL4 binding element andminimal TATA region of the 35S promoter of Cauliflower mosaic virus (CaMV), and these sequences are located upstream of LUC. To construct pBD vector, the coding region of GAL4 DNA-binding domain (DBD) was amplified from GAL4DBD vector (kindly provided by Dr. Jun-ping Gao), and ligated into pEAQ vector.

The transcriptional activity of MaWRKY26 was assayed with the re-constructed pBD carrying the full-length of *MaWRKY26* without the stop codon as effector (pBD-MaWRKY26). For the assay of the binding activity of MaWRKY26 and MaVQ5 to the promoters of JA biosynthetic genes, the promoters were cloned into pGreenII 0800-LUC double-reporter vector^70^, while *MaWRKY26* and *MaVQ5* were cloned into the pEAQ vector as effectors. All primers used for generating constructs for transient expression assay are listed in Supplementary Table S1.

The constructed reporter and effector plasmids were transiently expressed in tobacco (*Nicotiana benthamiana*) leaves as described above. The dual-luciferase assay kit (Promega) was used to analyze the transient expression in tobacco leaves 3 d after infiltration. Absolute LUC/REN was measured in a Luminoskan Ascent Microplate Luminometer (Thermo Scientific) according to the manufacturer’s instructions, with a 5 s delay and 15 s integrated measurements. The transactivation ability of MaWRKY26, and the binding activity of MaWRKY26 and MaVQ5 to JA biosynthesis gene promoters are indicated by the ratio of LUC to REN. At least six biological replicates were assayed for each combination.

### Statistical analysis

A completely randomized block design was performed in all experiments. Data were plotted in figures as means ± standard errors (S.E.). Statistical comparisons of the mean values were performed by one-way ANOVA, followed by Duncan’s multiple range test at the 0.05 or 0.01 confidence levels using the DPS7.05 software (Zhejiang University, Hangzhou, China).

## Additional Information

**How to cite this article**: Ye, Y.-J. *et al*. Banana fruit VQ motif-containing protein5 represses cold-responsive transcription factor MaWRKY26 involved in the regulation of JA biosynthetic genes. *Sci. Rep.* **6**, 23632; doi: 10.1038/srep23632 (2016).

## Supplementary Material

Supplementary Information
