# Supplementary material for: Banana fruit VQ motif-containing protein5 represses cold-responsive transcription factor MaWRKY26 involved in the regulation of JA biosynthetic genes
Source: Sci Rep. 2016 Mar 23;6:23632. doi: 10.1038/srep23632 (PMC4804309; doi:10.1038/srep23632)
Supplement: Supplementary Information [file srep23632-s1.pdf]

Banana fruit VQ motif-containing protein5 represses cold-responsive transcription factor MaWRKY26 involved in the regulation of JA biosynthesis genes

Yu-Jie Ye[1 #], Yun-Yi Xiao[1 #], Yan-Chao Han[1], Wei Shan[1], Zhong-Qi Fan[1], Qun-Gang Xu[1], Jian-Fei Kuang[1], Wang-Jin Lu[1], Prakash Lakshmanan[2], Jian-Ye Chen[1]*

[1]State Key Laboratory for Conservation and Utilization of Subtropical Agro-bioresources/Guangdong Key Laboratory for Postharvest Science, College of Horticultural Science, South China Agricultural University, Guangzhou 510642, China.

[2]Sugar Research Australia, 50 Meiers Road, Indooroopilly, Brisbane 4068, Queensland, Australia

[#]These authors contributed equally to this work.

*Corresponding should be address to Jian-Ye Chen (chenjianye@scau.edu.cn).

Supplementary Information

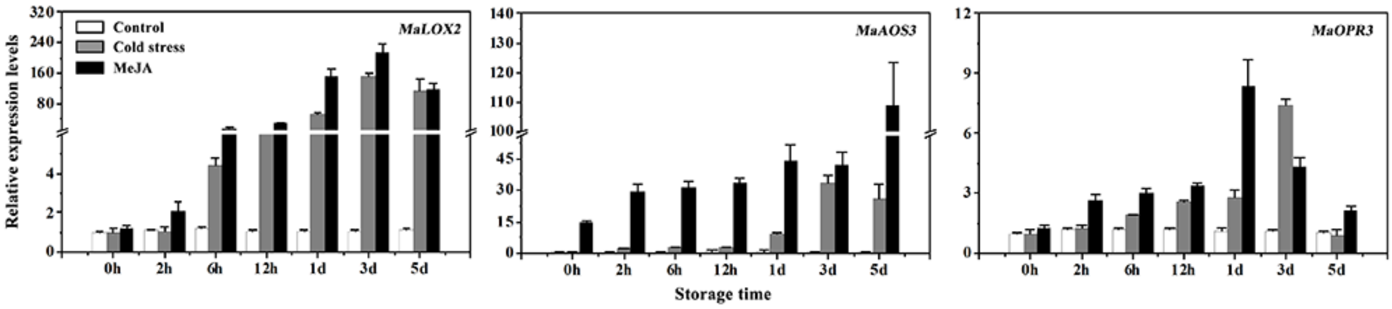

Supplementary Figure S1. Expressions of *MaLOX2*, *MaAOS3* and *MaOPR3* in MeJA-treated banana fruits during 5 d of storage at 7 °C. For cold stress, fruits without MeJA pretreatment were directly stored at 7 °C, whereas for non-cold stress control, fruits were directly stored at 22 °C. Expression level at different time points was expressed as a ratio relative to the harvest time (0 d of non-cold stress control), which was set at 1. Each value represents the means of three biological replicates, and vertical bars indicate the S.E.

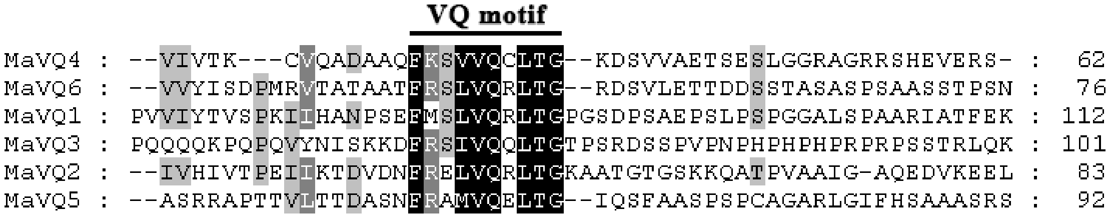

Supplementary Figure S2. Alignment of MaVQs proteins. The VQ motif is overlined.

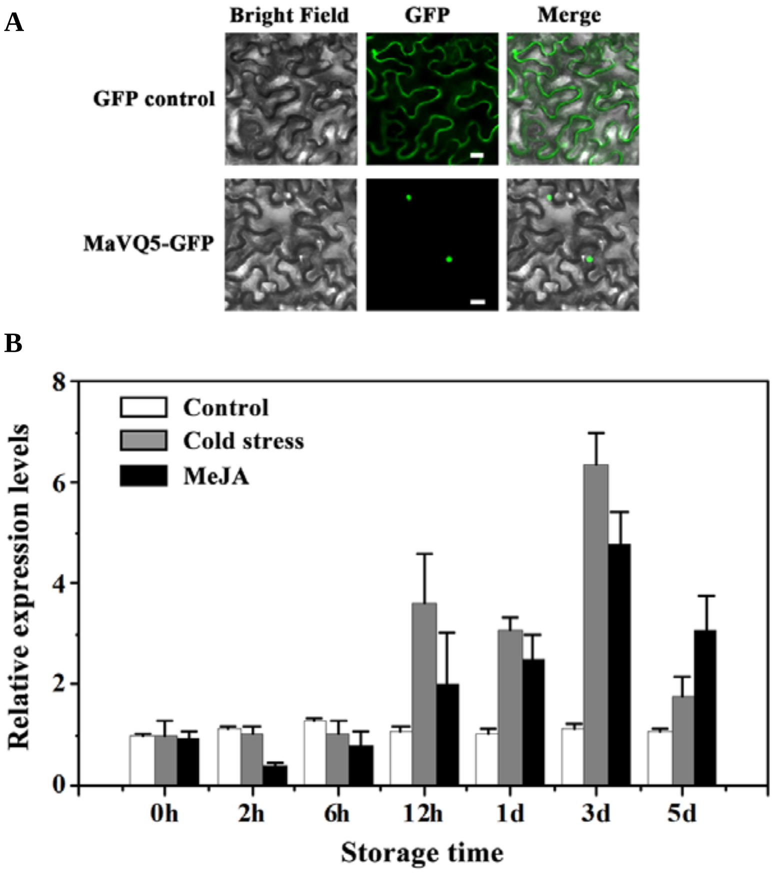

Supplementary Figure S3. Molecular characterization of MaVQ5. (A) Subcellular localization of MaVQ5 in tobacco leaves. MaVQ5 fused with the GFP or GFP positive control were infiltrated into tobacco leaves via *Agrobacterium tumefaciens* strain GV3101. After 48 h of the infiltration, GFP fluorescence was visualized using a fluorescence microscope. Bars, 30 μm. (B) Expression of *MaVQ5* in MeJA-treated banana fruits during 5 d of storage at 7 °C. For cold stress, fruits without MeJA pretreatment were directly stored at 7 °C, whereas for non-cold stress control, fruits were directly stored at 22 °C. Expression level at different time points was expressed as a ratio relative to the harvest time (0 d of non-cold stress control), which was set at 1. Each value represents the means of three biological replicates, and vertical bars indicate the S.E.

Supplementary Table S1. Summary of primers used in this study

Assay	Primer sequence	Restriction Site
Full length cloning	***MaVQ4-F:*** **ATGTCTTCCGACCACGCACG**	
	MaVQ4-R: **TCAATCGCTCAGCAACTCGAACA**	
	MaVQ5-F: **ATGGACTCAGCTAAGAGTAGCAG**	
	MaVQ5-R: **GCAATAACTCAATCTTCCTCCTCTC**	
	MaVQ6-F: **ATGAAGAGACTGGGCGTCCA**	
	MaVQ6-R: **CTACATGAATCCAGAAAAGCTCTCC**	
	MaWRKY26-F: **ATGGCTTCTTCCACGAGGAGCTT**	
	MaWRKY26-R: **TTAGTAGGGGAAGAGGGTAACATG**	
Subcellular localization	***MaVQ5-pEAQ-GFP-F:*** **TtctgcccaaattcgcgATGGACTCAGCTAAGAGTAGCA**	**NruI**
	MaVQ5-pEAQ-GFP-R: **TagtcataccggtcgcTCTACACCTCTTCTTTATCT**	**NruI**
	MaWRKY26-pEAQ-GFP-F: **TtctgcccaaattcgcgATGGCTTCTTCCACGAGGAGC**	**NruI**
	MaWRKY26-pEAQ-GFP-R: **TagtcataccggtcgcGTAGGGGAAGAGGGTAACAT**	**NruI**
RT-qPCR	***MaVQ5-F:*** **CTCTTCTACTTCCCCTGCTC**	
	MaVQ5-R: **GCTTTTGCTACTGGCTGAACGA**	
	MaWRKY26-F: **GAGGAGAACGAAAACCCGAAGTCA**	
	MaWRKY26-R: **CTCCTGGGATTTCGATTCGCC**	
	MaAOS3-F: **GTTCGACCGACCACAGGAGTT**	
	MaAOS3-R: **GTGAGCTTGACGGAGGAGC**	
	MaLOX2-F: **AGGAGCTGAAACAGGGTGCC**	
	MaLOX2-R: **TTCGGTGATAGCGGATTCAGGTG**	
	MaOPR3-F: **GAAGCGGTCGTCGGTGAGAT**	
	MaOPR3-R: **GGATGTGATACCTGCCATTGACG**	
Y1H assay	***MaAOC1- F:*** **TtgaattcgagctcggtaccCCATCATCCCGACATCAGCAC**	**KpnI**
	MaAOC1- R: **AtgcctcgaggtcgacCGCCGCTTCACATACTCTCG**	**SalI**
	MaAOC2- F: **TtgaattcgagctcggtaccCCTTCCTCCCAATCACCCAAG**	**KpnI**
	MaAOC2- R: **AtgcctcgaggtcgacGGCTTCCAGAGATGTGGTAGC**	**SalI**
	MaAOS2- F: **TtgaattcgagctcggtaccCTAGCTCCAAACTATCTTACAGC**	**KpnI**
	MaAOS2- R: **AtgcctcgaggtcgacCATCGGTGTTACAATCGAAGTAC**	**SalI**
	MaAOS3- F: **TtgaattcgagctcggtaccCCACGTGTACGCTTCCAGATTA**	**KpnI**
	MaAOS3- R: **AtgcctcgaggtcgacCGTCGCTTACTGTTGCTGATG**	**SalI**
	MaLOX1-F: **TtgaattcgagctcggtaccGGAGGACGGTGTGGCGAAT**	**KpnI**
	MaLOX1-R: **AtgcctcgaggtcgacCCGAACCCAAACGGATTCGA**	**SalI**
	MaLOX2-F: **TtgaattcgagctcggtaccGTAAGTGGCATTTACACCACTC**	**KpnI**
	Ma LOX2-R: **AtgcctcgaggtcgacCACACGCTGTATGGGTGTGA**	**SalI**
	Ma OPR1- F: **TtgaattcgagctcggtaccAAGGAGATCAAGAAGGGGCG**	**KpnI**
	Ma OPR1- R: **AtgcctcgaggtcgacGTCTCGGCCCGCCCAAAAG**	**SalI**
	Ma OPR2- F: **TtgaattcgagctcggtaccGGATGTCGTAGACTGAGCAAC**	**KpnI**
	Ma OPR2- R: **AtgcctcgaggtcgacGCTCAGAAACTTGGAGGAGAT**	**SalI**
	Ma OPR3- F: **TtgaattcgagctcggtaccGGGAGATGCGATCTGCACAT**	**KpnI**
	Ma OPR3- R: **AtgcctcgaggtcgacCTTACTAGCGCACACAAGAAG**	**SalI**
	MaWRKY26- F: **GgaggccagtgaattcATGGCTTCTTCCACGAGGAGCTT**	**EcoRI**
	MaWRKY26- R: **CgagctcgatggatccTTAGTAGGGGAAGAGGGTAACATG**	**BamHI**
	MaWRKY26-N-pGEX-F: **GgttccgcgtggatccATGGCTTCTTCCACGAGGAG**	**BamHI**
	***MaWRKY26-N-pGEX-R*:** **CagtcacgatgaattccaTTAGTAGGGGAAGAGGGTAAC**	**NotI**
	MaAOS3-Probe-PCR-F:	
	**TTTATTTTTTTGACCAATGGATTCGACTCCACGTGTCTATTTGACCGCTGTACACTTC**	
	MaAOS3-Probe-PCR-R:	
	**GAAGTGTACAGCGGTCAAATAGACACGTGGAGTCGAATCCATTGGTCAAAAAAATAAA**	
	MaAOS3-Mutant probe-PCR-F:	

**EMSA assay**	**TTTATTTTTAAAAAAAAATGGATTCGACTCCACGTGTCTATAAAAAAGCTGTACACTTC**<br>***MaAOS3-Mutant probe-PCR-R:***<br>**GAAGTGTACAGCTTTTTTATAGACACGTGGAGTCGAATCCATTTTTTTTTAAAAATAAA**<br>***MaLOX2-Probe-PCR-F:***<br>**GAGATCGCCATGTCCCTTTATTGGAGAAATTGACTTGGCAGATCGAACTCAACAATCA**<br>***MaLOX2-Probe-PCR-R:***<br>**TGATTGTTGAGTTCGATCTGCCAAGTCAATTTCTCCAATAAAGGGACATGGCGATCTC**<br>***MaLOX2-Mutant probe-PCR-F:***<br>**GAGATCGCCATGTCCCTTTATTGGAGAAAAAAAAAATGGCAGATCGAACTCAACAATCA**<br>***MaLOX2-Mutant probe-PCR-R:***<br>**TGATTGTTGAGTTCGATCTGCCATTTTTTTTTTCTCCAATAAAGGGACATGGCGATCTC**<br>***MaOPR3-Probe-PCR-F:***<br>**GGAGAGGAGAGGAGAGGGGGTTGAACTTTGACCACTTGAAAGCGTGGAACCTGCTTTG**<br>***MaOPR3-Probe-PCR-R:***<br>**CAAAGCAGGTTCCACGCTTTCAAGTGGTCAAAGTTCAACCCCCTCTCCTCTCCTCTCC**<br>***MaOPR3-Mutant probe-PCR-F:***<br>**GGAGAGGAGAGGAGAGGGGGTTGAACAAAAAAAACTTGAAAGCGTGGAACCTGCTTTG**<br>***MaOPR3-Mutant probe-PCR-R:***<br>**CAAAGCAGGTTCCACGCTTTCAAGTTTTTTTTGTTCAACCCCCTCTCCTCTCCTCTCC**	
**Y2H assay**	***MaWRKY26-Y2H-F:*****GgaggccagtgaattcATGGCTTCTTCCACGAGGAGCTT**<br>***MaWRKY26-Y2H-R:*****CgagctcgatggatccTTAGTAGGGGAAGAGGGTAACATG**<br>***MaVQ4-Y2H-F:*****CatggaggccgaattcATGTCTTCCGACCACGCACG**<br>***MaVQ4-Y2H-R:*****GccgctgcaggtcgacgTCAATCGCTCAGCAACTCGAAC**<br>***MaVQ5-Y2H-F:*****CatggaggccgaattcATGGACTCAGCTAAGAGTAGCA**<br>***MaVQ5-Y2H-R:*****GccgctgcaggtcgacgCTATCTACACCTCTTCTTTATCTC**<br>***MaVQ6-Y2H-F:*****CatggaggccgaattcATGAAGAGACTGGGCGTCCA**<br>***MaVQ6-Y2H-R:*****GccgctgcaggtcgacgCTACATGAATCCAGAAAAGCTC**	**EcoRI**<br>**BamHI**<br>**EcoRI**<br>**SalI**<br>**EcoRI**<br>**SalI**<br>**EcoRI**<br>**SalI**
**BiFC assay**	***MaVQ5-BiFC-N-F:*****TtctgcccaaattcgcgATGGACTCAGCTAAGAGTAGCA**<br>***MaVQ5-BiFC-N-R:*****TcaccataccggtcgcTCTACACCTCTTCTTTATCT**<br>***MaVQ5-BiFC-C-F:*****TtctgcccaaattcgcgATGGACTCAGCTAAGAGTAGCA**<br>***MaVQ5-BiFC-C-R:*****CgctgccaccggtcgcTCTACACCTCTTCTTTATCT**<br>***MaWRKY26-BiFC-N-F:*****TtctgcccaaattcgcgATGGCTTCTTCCACGAGGAGC**<br>***MaWRKY26-BiFC-N-R:*****TcaccataccggtcgcGTAGGGGAAGAGGGTAACAT**<br>***MaWRKY26-BiFC-C-F:*****TtctgcccaaattcgcgATGGCTTCTTCCACGAGGAGC**<br>***MaWRKY26-BiFC-C-R:*****CgctgccaccggtcgcGTAGGGGAAGAGGGTAACAT**	***AgeI***<br>***AgeI***<br>***AgeI***<br>***AgeI***<br>***AgeI***<br>***AgeI***<br>***AgeI***<br>***AgeI***
**Dual-luciferase transient expression assay**	***MaWRKY26-xPEAQ-F:*****CgcgaccggtgtcgacATGATGGCTTCTTCCACGAGGAG**<br>***MaWRKY26-xPEAQ-R:*****AgttaaaggcctcgagTTAGTAGGGGAAGAGGGTAAC**<br>***MaVQ5-xPEAQ-F:*****CgcgaccggtgtcgacATGGACTCAGCTAAGAGTAG**<br>***MaVQ5-xPEAQ-R:*****AgttaaaggcctcgagCTATCTACACCTCTTCTTTATC**<br>***MaAOS3-F:*****CggtatcgataagcttGTGCGGACACCACATAGCAC**<br>***MaAOS3-R:*****TagaactagtggatccCGTCGCTTACTGTTGCTGATG**<br>***MaLOX2-F:*****CggtatcgataagcttGTAAGTGGCATTTACACCACTC**<br>***MaLOX2-R:*****TagaactagtggatccCACACGCTGTATGGGTGTGA**<br>***MaOPR3-F:*****CggtatcgataagcttGGAGATGCGATCTGCACATC**<br>***MaOPR3-R:*****TagaactagtggatccTCTTACTAGCGCACACAAGAAG**	**SalI**<br>**XhoI**<br>**SalI**<br>**XhoI**<br>**HindIII**<br>**BamHI**<br>**HindIII**<br>**BamHI**<br>**HindIII**<br>**BamHI**

Supplementary Text S1. Nucleotide sequences of the promoter of JA biosynthetic genes.

W-box (TTGAC) is indicated in box. Translation start site (ATG) is shown in red.

> *MaLOX1* (GSMUA_Achr9T16020_001)

AGATCAGTCTTAGGGTGTTTAAGCGATTACGGATGGATGCAACTCGTGCGTACTTCAGTCCTATAAATCGTGATCATCAAGTACGTAAA
TGATTCCATCTGCTTCGTATTTACATTTTTTTATAGGAGAAAGCTAATCTCCAACTTTGTCGTGGAGCATCATGACCTTCTCTTGTCA
GCATCACCATCTCCTTCTGACGACCGGACTTTATATCATATATCTTTTGCCGACTGCTTTATCGAACAGTTGCGTTGACCAATTGGTTC
CGATTTGACAATTCTACGCTACCATAGAACAAGGGGTTTATTTACAAGGCATTAAAGAAGACATATATGCAACCCATAAACCATACTAA
TACATAAAACGAAGTGCAACATCAATTATGTTTGATATTAGCCTTGGCGAAGTATGCATCCCCACACTCGTACACAGCTGCCGGTCGTC
CATGGCGGATGAAAGCAACGCGACTAACCAGTCAATTCGTTAGGCAGGTTTTTATGTGAACACATGACTTTCTAGAGCTCCATAATTCA
AACTCGAGTTTCATGGTTGATTCATTCGAGCGAGAACCGAATCAAAATCGAATCCAGTTTAAATTTCTTAAAATTGAAATCGAACCTTT
TATTCTGGTTTTTAGCTTCTTTTGCCCTTGAAAAGGGAGGGCGGTGCTGCTGCATTGGTCTCCAAATTAGTAGCTGCTTTCCTTCCTCC
TCCTCCTCCTCCGCCTCCTCTCTTCTTTGTTATATACACATAGATACATAGAAGACAGGTAGAACACGCGATCTCCCTTCTTTTCTTAC
TTTGTTTCTTCTTCTGTACTGCTCAGATTAATAATGTAAGAGCGTAGAGAAAAAAGTAGTGTGGCGATTAAGACAGTCCCTTTCATTTT
TGTGAGGATAGCACAGAACTTCCATCTCTTTCCTTCCAGTAAGGGACGAAAGGATCCCAATAAACCTCAAGTTCTAAAGTCAACGTTAG
CGACGAACTATTCCTCCAACGAAGAGCTCGTTGTCTTATTTTGTTTGATCCACTGTTGCTGCTTTCACATACCTACGCAACAGCTAATA
AGTTCGATACACGTGGCTTCCTATTCCCTTAGCTTTGGAATCATGTGGGTTATTATGCCCTTTATAAACCCACCCAAATTGTTGTCCTC
CCATCGGTTATTTGATATCCATCAATTAATTATTATTATTATGACGTCGGAATCCCATCCGAGCGTCGGAGGACGGTGTGGCGAATTGG
GCTGCGTTACATGCGTTTAGGTGTTTGACCACACGTGTGGCGGATGTGGGTCAACACGGATCTCCGTTCAAATTGAAGGAATCGCACGA
AGGAAAAAACCATTCGCACGTGGAGCAGTGGGGGCGCCCTATTCATTCGGACCCCACCAAGTTCGCATGCCCTCATTCTTTCACCATAT
GACAGTGGTCTCCCCCATTAACTCAGTGGGTGAGAGGCGACGTGGCCACCATCACACGCTCCCAAAGCTACTTTGTTATGTATACTTTT
TCCTCTTTTTCTTGGCCACGTCAGCACGGTGTGAACAACGAGTTACTTGTGCGGAACACCAGCAATGATTGATTACTGCTTACGTGGAA
TATTCAGGAATATCTGGCAATTTCACCCAATTGGCATCAGATCGAATCCGTTTGGGTTCGGTTCGAAATCATTCTATCTACTCGATTCA
ATTTTGATTTTGGACCGGTTCAATTTCGGTTCAAGCCGTGGGGGTCAAATACGTAGATGAAATCCGATCTATCTGATTTTTTTGGGAGG
TAAATAAATAAGCTAAGATTTTTCCGAGCTAAATAAATCATAGTATCCTTTTACACTTGGGATAATGGAAACCCATACGATTTCTACAT
GTTCTGTTTTGTGTTCCGTCATGCTTACGGGACTGGATTGGTATTTTAGGATTTGTGGTGGGCATCGGCGGTCCTTTGAAACGGACGTC
GTTGCCGCGAAGCTGCCATTTGGCCCTTATTTATCGCGAGGGAGAAAGCGTGTCCGTGTGCAATGCCCATTTTACCCACTCGCCAGCCC
CTATATAGGACTTGCCCCACCTTCCCCTGTTCGACCAAGGAGAGTCACCAAAGAAGGGGAGGAATTCAAGCGGACGGGAAACCAAGTCT
TGCGCTTCTGCCCAAAAATCTACTGTTTCCGTCGGGAAGTTGAGATTTTGGGAAGGGGACGATATTGGATG

> *MaLOX2* (GSMUA_Achr10T17530_001)

GAGTCAAAAGGTAGGATGACACGCCCATGGACGTAACGGAGGGATCACTCCATGACTAGTCTCCCGATCTCGGAACTTGGATCTTCAAC
CACGGAAAAGCCCAGCCATGGCGACGCGGCGTCAGCCACCAACTCCACCCCACCCCGCAAGTCCAGCTCGTAGTTTGAAGTCAAAGTCC
CAATTCAACGCCTTCGATGGAGCTCTCCAAATCCACGTACGTCTCGTGGAACCGTCCTCCAAACCAACGGCGTGCTGCCAGGCTTCGTC
TCTTGTGATCGAGCTCCGTGGCTGTTGGATGGGCAGGGAGGCCTTTGAGGCCCAGTCAACGGAGGAGGACGATTCAACATGACCGTATC
GCCCTGCCCTTCCCGGAAGCTCGCCGCAGATGAGACCGGCGAACGCAGATCCATCAAGAACAAAAGCGAGGGCGGTCGTCTCTACAGCT
TTGTTTCCTTTGACATAGAGACACATTTGAAGGTGTGTTTGTCAGGGACGACTCGATCGATCCGTCTTAATCATAGATTGCATGTGTAA
CAATCTGAAAATAATTACACGTCAGTCATAGATCTAGTTAATTTTGTCTGAATCGTACAGCATCATTTATGTTCAGCGATTAATTTTCT
CAAAATATTTTATGATCCTTTAGCACCGGACGAGTTTAACTCGAGATTCTATGAACAATTATTTTAGTCAACACTTGATAGATAATATA
AATTATAAAAATAAATTTCTTAAACTCTGAACAGTCACATAACAAAAGATCTAAGATCATCTATCACCATCAAAAGTTTCTGCTAAAAT
CGGATGTTCGATTCAATTTCTCTTTTAATTTAGGATTATATGAAAAGTAAGGTTATTAAGAATGATAAATTAATCAATTTTATAATAGA
TTGGATAAAAAAAAAATAGATTATAATATGAATAAAATATTTTCTGTTTTTTTTTACCGATGTTACTATATTTAAAGGATAAGAGATTGT
CTGAGCATCAATCATTAGAAATGTTACATTCCAGAGAAGAACAGAATAAGAAAAAATAATTTGCAAGTGACAAGTGGTAAAGACAAGAT
TTGAGCTATAATTTTTCTATCAACCATCAAAGTCAGTCTAAACAAAACTAATAAATTAAGCCAAAACACTTTATATTTACAAAAAAACA

AAGAGAGCACTCTTCCCACGCATGAAGATCAACATCTAGAAGGGTTCCTAACGTGCGACCTCTGTAATTATATTTGCATTCACCTTTTA
TAGTTCATCATTATTTTAAATCACTCATGATTATAAAAAAAAAATTATATTAAAAAATATCCATGGAATATTTTTTGTTCCGATGATTA
TGTCGACATGATGCGTCCTTTGGTTAAGCTTTTATTATTTGTGCTAGCTATGCATCGACGAAGACGAAGACGAAGACGAAGACGAATAT
CCTCGTTTCACTTTCAATTTCATATTTTCAAATACTACTCACGAAATTAACTATCACTCATTGTCATACAAGAAATGTATGAAAAAGGA
TTAGAGTGCATAGTCTTCTCCCTTTAAATCGAATATTTGGGTGACAACAACGGGAAGCTAATTAGGCGAGTCATTAAAGAATTAGACTT
CCGATGACTTCAATTATTATAGAGAAATTTCGGTATTTAAATGATTGAAAAGAAGATAGTAAGTGGCATTTACACCACTCATTAGTTTG
TGGTTGAGATAATAAATGATAAGTTTTATGTCATAAAAAATAACACCACGAATTCGTTTTTTTACTTGAACGTCACGAGATCGCCATGT
CCCTTTATTGGAGAAA**TTGACT**TGGCAGATCGAACTCAACAATCACACCAAGCAAGATTGAATTCTATTGATGTTGTCCTGTGAGCAAC
TAAAAATATGATAGCAAGAATCAAACAAACAAGTTTGATATCTCCTCCTGTCCTTATGAAGTACCTACGATGAGGAAATCATCCCCATC
ATGTTCCAATGATCGAGTTGCGGGATTCAGTGCGAAAGTAGTACTGCTTCTGCCATTGATATGCCATTTGAACACCACAGATGGGCATG
CCGGTCCTTCGAGCTGCAGAAAATGAACCCCACTACCTGGAAACATCCTTCTTTTCCTCACGG**TTGACT**TTGTTCTACCATCACGCAAT
CGATCACACCCATACAGCGTGTGTTTATGAGCTATAAACGCCAAGAAATCAAACAAGCAACCTGCACGAGGTAGAAAAGTAGAAACCAA
GGGGCCGCCTGCCACCCCTCCCTTCTTATATTTGCCAACCCATCCTTAGATCACACCGCAATAATTGTTGTCGCAACAAAACAGAGCAG
GAAGAAGAAGAAAGCTTCCCCCCCCCCCCCTTCTTTCTCCTGAAACTTTGCTCGAGTGACTACTTTGATCTTTAGGAAGAAAAGAAGGG
AGGGAATG

>*MaAOS1* (GSMUA_Achr6T13520_001)
AAATGAACTACTGGCATCTTGCTAGCTCCAAACTATCTTACAGCTTTCGTCTCTTTTGGCTTTTGGAGGAAGGGGATGAGC**TTGACT**TT
CACAAGGACTCTTATTGCAAATTATCTTTGGCTTATTTGGAAACATATGAATGAAACCAAATCATCCATTCCCCCTTCACTTCACTCTT
TATTGAGATAGCTCTTTTCCTTCTCTGCAGGATCAAGAACCCTAGTGGCAGGCACTTCCTCCTCCACAACACTGAAGACGTCAGAGCTG
TCTCACTGCATAGGCTCAAAGATCCTTGAAGTCTTCGCGGATTCTATGACCCTTGGGAACATCCCAACCAAACGTTCCAACATCCTGTA
CCCCTTCTGCAAGGATATTTTGTACTTGGCTTCTGCTGTGCATGTCACTCACTGTAGTCATATCAGCAGAACAATCAACACAATAACTC
ACAGCATAATTAATTTTGCCAGACTGGCAATTCTTTTATTTTTCTTAGAGGCGACCCACCACCACCACCACCTAATATCCTGTGTG
GTTTCTTTTTAGACAAGTAGGACCCACTGACGTTAGTGATTGACGAGGCATGTCGAGTGTATTATTTCTTTCAATCATGACAATCATTA
TTTATCGTATCATTTGTACTTCGATTGTAACACCGATGTACTCGTAATTCAACCGTGTTATTTTACCTGTCACCATCAGAATTACGTTC
CTAATTATCCCACGTCATCTTAAGTATTATTCCGATCACTATCAACCCGTGAACGTTATCCAATAATCCGGACGCTGAGTTGTAGTGAT
GTGATTCATCCAAATCACATGTCTGATCTCCGAATTCTATCCCAGCACGGCACGCCACGTCAAAAGAGAAATCGAATCATTGTTCTGCG
GTGGGTTGGTAACTAACGCCGCGCCGTACGGTTCACGAGAGCCGTTGGATGGCGTCCGTTCACGTGAATCCATCGGTCGGTCGGATCCA
CGAAAAGGAACGCGTCTCATAATATCTCCGCTGTAGCTGCTCAATTATTGGACCATTCTTTAGCCGCAAAAAGACAAATAGAGACGCAG
CAGAGAGCGCAGTTAACTTGCGGCAGGTCGATACCGACCGACCGCTATATGAGCACGACATGAAGTGAGAGGAGGAGGAGAAGAAGAAG
AAGAAGAAGAAGAAGAGAGGGAAGGTACGGATG

>*MaAOS3* (GSMUA_Achr4T32370_001)
ATGTACTAATGGAGAAAGATTTTTTGAGTGCGGACACCACATAGCACGTGGTGTTGGACGGTGGCGTGCGGGGATCACGATTCCAATCC
CAAAATCCCTCTCTCGCGTGCGAACCCCCGCCCCCCCCCTCCAAAGCCCACGTGTACGCTTCCAGATTATTGTAACCTTTTATCAAAGA
AAATAAAGCATAAAAATATGGACACAAAGAGAGAGAGAGAGAGAGTTTGTTTTTCTTTGTTTTTTCTTTTT**TTGACC**AATGGATTCGAC
TCCACGTGTCTAT**TTGACC**GCTGTACACTTCACGTTTCCATCCTTCGATATCG**TTGACT**ATTGACGTGGCACACCGGCATGCGCCCACC
ACACTAACTCGTGGCTCCGACCATTTCCCATTTCCTTCTCACTTCCCCGTAGCCGCCTGCTTTTGGTGATTTTATTTTTTTGCTTATTA
TTATTTCTCTCTCTCTCTCTCTCTCTCTCTTTTATTTCTTAGTTTAATAGATTATTTATTAATAATACTATTATAATGACGCCTTCTGCGT
CTATAAA**TTGACT**CCCTCCTCTGCCTCCGTCGTCGACGACTCCCTTCGTCTCTCTTCCTACTTCTCTTGCTTCGCATCAGCAACAGCAA
ACGACGATG

>*MaAOC1* (GSMUA_Achr4T10390_001)
GGACACCCTGAGCCGCTACCATTCCTACCAGGAGACAGACAGTGACCATCATCCCGACATCAGCACCATGACCTGTGGTCGATCAACTT
CGACGACCCGTCAAGCTGATCT**TTGACT**CCCAACA**TTGACC**CGTGGAGACATTGGTATCATCTGGTATTCGTAGTTTTAGAGTACGTAA
CTGCCCCTCTTTGTCTTCTTCTTCTTCTTTGTGTTATACATACATATTACTCCCCGAGAAAAAGAGCGACACATCTTAGCCGACGTGTC

CTTCCATAAGTCGCCTTAATCATGAATTAATGGATATATACAAATATTTCATCTATATATATATTATGATTATTCCATTGATTTTAATG
AGCTATCGATCAAATCGATCATGGAAAGTTTCTAGAAGATCCATTTAACTGTCCGTTTCGAGTTGTGCACCCACACTCCAATCATTCGT
TGCCCTCCTTCGACGACCACGTCACTGCTTACGTTCACGAGAGTATGTGAAGCGGCGTTACATATACCGCGGTGCGGCAAGATAAGCTC
AGATTGCGGCGAGCAAAGGCGATGAAGCAAACGGTGGTTTTGTACGCAGTGCCGGCGATGGGCCACTTGGTGCCCATGGTGGAGCTGGC
CAAGCTCTTCGTCCTCCACGACTTCTCCGTCGCTGTCGTCCTCATGCACACGCCCGTAAAACATCCCTCCGTGGACCCCTTCGTCGCCC
GCGTCTCCTCCGCCTACCCCTCCATCTCCTTCCACCAGCTCCCGCCGGCCGCCTCACTCCCCGATACCCCGCTCCCCCGCTTCTTGGAC
CTCGTTCTCCCCAACAACCCCCAGCTCCTGTACTTCCTTGAAGCCCGTTCCCACACCTCCGACGTCCGCGCCGTCGTCCTCGACTTCTT
CTGCACCGGTGCCCTGGCCGTCACCGCCAACCTCCGCCTCCCTTCCTACTTCTTCTTCGCCTCCTGCGCCGCCGTTCTCGCCGCCTTCC
TCTATCTTCCGACCCTCTACGCCACCGCCGACATCGACTTAAAGGCCCTCGGGGACTCACCGCTCCACTTCCCAGGGCTGCCCCCCGTC
CCCGCCTCCGACATGCCTCGCAACATGATCGACCGCGATGAAGACTACTTCAAGAGGATGATACGTGCCTTAGAGAGCCTGCCGAACGC
CGACGGCATCCTGGTCAATTCGTTCGAGTCCCTGGAGGCGGAGGCCGTCCGGGTCCTTCGGGACGGGGCCTGCATTCCCGGTCGTCGGA
TGCCACCGGTTTACTGTATCGGGCCGTTGATCGCCGACGGGAGCAGGGACGTCGGAGGAGAAAAGATGGAGAAGGCTGAGTGCGCGTCG
TGGTTGGACGAGCAACCGCGCGGGAGAGTAGTGTTCCTCTGCTTCGGTAGCATGGGGACGTTCTCCGCGGAGCAGCTCAAGGAGATCGC
GGCTGGCCTGGAGAGGAGCGGACAGCGGTTCCTTTGGGTGGTGCGGGCTCCGCGGAGCGAGAGCCAGGGGCCGCAGGGATGGGGGCTGC
AGTCAGAGCCGGACTTGGAGGCCCTGTTTCCGGAAGGCTTCTTGGAGCGGACAAAGCAGAGAGGATTCCTGGCGAAGTCGTGGGCACCG
CAGGTGGAGGTGCTCAACCACGAGGCGGTGGGGGGGTTCGTAACGCACTGCGGGTGGAACTCGGTGCTGGAGGCGATCACGGCCGGGGT
GCCGATGATCGGGTGGCCACTGTACGCGGAGCAGGGGATGAACAAGGTGCTCCTGGTGGAGCAGATGCGGGTGGCGGTGGCGATGGAGG
GCTGCGCCAAGGAGCTGGTGGCGGCGGAGGAGGTTGAGGCCCGGATCAGGTGGCTGATGGAGTCGGAGGGGGGGCGGGACCTGAGGGCA
CGGGCGGTGGCGACGAAGCAGAGGGCGGCGGAGGCGATAAGGGAAGCCGGGTCGTCTCATCAGGCGTGGCTGGACGTAGTGAAGACCTT
GAGGAATGGAAGCACGTCACCACTTCGAACCACAGGATTGACGAGTGAGGACCACCTCAAGGTGCCGTGCGATTGATGACATCCGCACC
TGTCGGTTGAGGAGTAGTGGACTCCCCATGTGATGAAGCAATTCAATGGTTTGTGGACCTTTGTTGGAAAACCTAAAGATAAATATTAA
AATATTTTGAATAAGTTAAAAATTTAGTCAAACAATTAAATTATTAATTAATTTATTTCTTAATAAATAATGTGATCCTTAAAAGTAAA
TAATTTAAATTTTATTTTTATATGTGAATAATAAAAAATATTATAATTTTACTTTACGTATAAAAGTAAAATAAAAATAAACTAAAAAA
TATATTATTGTTTACCTGAGCTAACTTTCAAAATTTTAAGAGCAGCTCATTTATTAGCTTATTAATTTTTATATGTATATTAATTGTTT
TTTTCCTCGCTTCTTTTCCATATATTCAACCCACACCTTTCACCCCCCCGCCCACCCCGCCCCGCCCAAAAAAAAACACTAATAATAAA
ACGGAGTCTCCTCAAACATG

>*MaAOC2* (GSMUA_Achr3T31910_001)
CTTTGCTTGCTAGTTAAGTACTTTTCCTCGATCAGTTTACCTTCCTCCCAATCACCCAAGTCTGCGCATGCAAGAATTACGTTGACCAA
AATGGATCTGGTAGGAGGAGTTTGTGTGTTCATGATCATCCATTCGAACAGTTTGAGGGCATCATATGGCCGCTTGTGTTGGACATATC
CACCAATCATGGTACTCCATGAGACGGCATCCTTAAACACCATCCTGTCAAATAATTTGCATGCCACACCCAAGCAGTCGCAGTTGGTG
TACATTTCAATTAGAGAATTCAAGACAAACAAACTGGCCTCAAAACCAAGCTTGATTGCATCAGCATGTATGGCCTCTCCGTCCTTGGG
AGAACCAGAACGCCCACAGCCGGAGATTAAGCTAACCAGAGTAGCCTCGCTGGGTAGAACTCCTTGGCCAAGCCTCATGACATTAAACA
AACTGACAGCCTCGTAGAAGGATCCAACTCTCGAGTACCCCGCGATCATGGCGTTCCAAGCTACCACATCTCTGGAAGCCATTCCATCA
AACACCTTTCGAGCGGATCCAATCTCCCCGGCTTTACCGTGCATATCAAGAAGCGCCGTCCCCACCATGAGATCGGAATCGAGCCCTTT
GGCCCGAGCATGTCCTCCAACTCTCGAACCCAAATCGAATTCGGACAAATGGGTGCAAGCCGTGATGACGAGGTGAAAGGTCACCCTGT
CCGGCTCGCACTGCGCCAGAGGCCGCGCCGCCACCATTTCACGATAGACGTGCAAGGCCGACCGAGGCCGGCCGTTGTTGAGGTATCCT
CTAATAAGAGAATTCCAGAGGAGGGTCTTCGACGGTCCCCCTCGTCGGCCATCGTCGAAGACTAAGCGGGCGGTTGCGGGGTCACCGAA
ATGGAAGTATCGGGCGATAAGGAGGCTGGCTACGTACAGGTTGTGACGGAGTCCGAAGGCGATGGCTCGGCCATGGAGGCCCCGGAGAT
GGTGGGCGGAGCAGCAGCGGGAGATGGCGTGAGCGAGCGCTTCTTTGAGGTCTTGTGGGCTGCGCTGAGCCTGGCAGTACAACCGCGTC
GTCAAGGTCATCAGAGGCGCGGAATTTAGTAGTAGGCGCGGAGAGGCGGACCCGACGAGAGCCGTTGATTTAAGAGATCCAGCCGAACC
GGACATGGGTCTGATGGAACCTACCCTCGTCCGACACAACCGGGCTAACGGGTCGGATCGTGTGGATCCGACAAATAGGATTGTAATCA
GACATTGTCTACACGTGCAAAACATGTGCAGAACTACCAGTAAACAATGGGGATTAAGTTGGTTAGATTGATCGGTAGCAGAAGAGAGC
GAGTGGCAGTACACTAATCAATCCGTGTGCGTTTCGCAGCGGAGTGAGACTTGGTGTGGAGGTCCGCCACCACGGGCTGCCTGTGCCAG
ACATG

>*MaOPR2* (GSMUA_Achr2T20790_001)

TTGAGTCATTCTTTAATAATTTTAAATATTAAAATCTAATTTGATGAGATGAGTCGAATTATGATCTAACTTTAGTCAAGTTGAT
CTGTCAGACCGGATCTCATGTGGTCATGTATAACCTAGTTCATGTGGCCAAGTACAACCTAGCTCATGTAACTAAGGATGACTCA
ACATATGTGACCTAGTACAAGCCAACGCAACGTATGACCCACCCATGTGGGTAGGTATGATCCAGTCTATGTGATCATGTATGAC
TCAGCTCATGTGGTAAGGTACGACATAACCATGTGGCCAAGCATGGGTCAGCTCATGCGACTAGGTATAACTCAGTCCATGTGAT
CAGATACGATCCCAATTCATGTGTCTATGTACGACCAACTCATGAACAAAGCATGGCCTAATTCACTAATAAAACTCTGCCTAAC
TTATAAGTGATACTTAGTCTAGTCTATAAAGTTAAGCTTGCCCAACTATGCGA**TTGACA**TTAGACACATCGTCGCTCCTTTATCT
ATCCCGTTGTACTTAACTCAATTTACTTGGAATAGGTATCTTGTGACTTGTTGAAGACTTAGGTGGATTAGTTCGATTTATATTA
GCGTTATATGATATAGTTTCGATTAAATGTATGCCACTTATTATTATTATAGTAATAATATCTGGAATCGGTTAGCACACCTAAG
TTGGATCAATCTCAATCGAAGAGAAGGAGGGTGGAGATGAGAGAATTGGAGGGGAATCGAATTGGTAGGGGTTGAAGAGGATCCG
AATCTTCTACATGGATGTCGTAGACTGAGCAACGAAAAGGAGGCGATGCTTTCTCAGTGACGGCTATGGCTAGCCGCCTGTCTTG
GTC**TTGACC**AAAGGTGCTCAAACTAGAATGTGGAGGCCTCCATGGCAGACTCTTCCTTTTCCTTTGGCACGTTTGTTGAAGGTTA
GCGACGTCGCTCGTTTTCTCCATCATCATATCAGTGATGGAGGGTTGAGATGAATCATCACATCATCAATTACCATCATCAATTA
CCATATCAGTGATGGAAGGTTAGCGACGTCGCTCGTTTCCT**TTGACC**ATCGAACGCACGCAGTGACGGTAGAGTTACGCCGGTAG
TCCTGTAGCAATCAATGCGCGTGAAGTAGAAAAGAAGAAATCGGACTAGGCTACTCCTCAGTCTCAAGCTTCACCCTTGCATTCC
TACAAATACCAAGCACTCATCCCCATCCTCTGCACGCACAGTCAGGATCCACTTGGTTTATATCTCCTCCAAGTTTCTGAGCATG

>*MaOPR3* (GSMUA_Achr2T20800_001)

ATCAAACACAATTATGTTTATTTATTTAGTGTACATACTTAACGAAATCTTACGGGATTTTGATCATACACCTCTTTGAGTCTTT
ATGATTTGTTCGTCAAAACATCCCATAGGGAGTAATTAAGGATCGGACTCTCCCACAAGTGGAGAAGGCTAGGCACTAGCTTAAA
GAGGTCAGGGGGTGGTGTTGCTAAGGGTGTAATAGGAGCGTAAAGCCACTTATACCTAGCCCCCGAATGTCAAAGCTTGAGGGGC
GCTTTTGATGAGGTAAGTATGAGGCTAGCCTTTGTCCCTTGGGAGAGGGAGAAGGGGTTCTCCTTGTCTTAAGCATTCATTGGGA
AGAGTAATAGAAGGGTGTGCTTGTGAAA**TTTGACC**CTCCTTCTAATACTAAAATGATGGTTTATTGATATCGTTGATTGATGAGT
CAGCACGGTTGGTTTACTTCCAAATGCATAAGTTTATCCGGATGCCTCTTTCTAGCTGACATGGGAGACTTAATGAGCGACCTAG
TCGGAAGTGCTCTTGTCGATGAGAACCTAGTTCGGGATGACCAGATGAATGGTTCGGGATCTTCTTAGTTGCAAGGTTACTTCTC
TGGGCCGGGTCATCTAAGTGTCCTCGAGAGATGATTTATCTTGTGGTTGTGTGTCTATGTCTTAACGATGAAGTTACTCGCTCTT
CGTCGATGATCTAGCATGACATGTCAACGTCGAGATTGCTCGACACAAACCCTTCTATGCTTAAGTCAATGAAAGAGTGAGTAGT
CGTGTAAATTGTATGATTATATTAGTGTACAGAGAGTTCTCCCTCGAGCATAACCTGATAGTTTTTTTTATACCTAATTCCGAG
ACCTTTCTTGTTGATCAGTTATAATTGATCTCGGTAAGGTGATGATTATTGAAATGCACTTTCTGCTATTTTGGGTAGGTGATAT
GAAGTCTTACTTAGATGAGCTGACATCCGACAAAATGACCCATATCAAATCGATTCCCATCCAATTCTCTCATCTCTACCCTCCG
TCTCTTCGATCCTCAATCTCAACCATCGATTGAGATTGATCAGACTCGCGGTGTGCTAACTGATACCAGATATTATTACTATAAT
AATAATAATAATAATAATAATAAACCAATACCTGATATTATTACTATAATAATAATAAGTGGCGTGTCTTCAATCAAAGACC
GTGTGGTGGTTGACGATGACATTTGCAATGACGTCAGAAGGAATATGATTACGTGGGGCGGCTATTCCAATTGCGCGTACGTGAA
AGCCTCTGCAAGAAGAGAATACGTAGGCAGATACGATACGCACACAAACTTTTCAGGGAGATGCGATCTGCACATCACTACCAAC
AATTATTTGATTCTGAAGCCAAGCTTTGTAGCCTCCACGCTTGAAGCGACGGCAAGGGAATCATCCAGGAGAGGAGAGGAGAGGA
GAGGGGGTTGAAC**TTTGACC**ACTTGAAAGCGTGGAACCTGCTTTGTTAGACCAGAAACATTGCACTGTAGAGACGGAGAGCGCAA
CAAGTGCACGTTCTTGTTTGCTCTGTCAACCTCAGGCCTCCTTCCCTGCATCTACACATACCCAGCAGCCACCGCATCCTCTGAG
CACCAAGGCAGATAGTTAGAGCAAAAGCAAGGGGAAAGGGAGAAGAAGAGGAAAGAGAAAGAGCATCCACTTCTTGTGTGCGCTA
GTAAGAATG
